# Regulation of AR mRNA translation in response to acute AR pathway inhibition

**DOI:** 10.1093/nar/gkab1247

**Published:** 2021-12-23

**Authors:** Syam Prakash Somasekharan, Neetu Saxena, Fan Zhang, Eliana Beraldi, Jia Ni Huang, Christina Gentle, Ladan Fazli, Marisa Thi, Poul H Sorensen, Martin Gleave

**Affiliations:** Department of Urologic Sciences, University of British Columbia, Vancouver Prostate Centre, Vancouver, Canada; Department of Urologic Sciences, University of British Columbia, Vancouver Prostate Centre, Vancouver, Canada; Department of Urologic Sciences, University of British Columbia, Vancouver Prostate Centre, Vancouver, Canada; Department of Urologic Sciences, University of British Columbia, Vancouver Prostate Centre, Vancouver, Canada; Department of Urologic Sciences, University of British Columbia, Vancouver Prostate Centre, Vancouver, Canada; Department of Urologic Sciences, University of British Columbia, Vancouver Prostate Centre, Vancouver, Canada; Department of Urologic Sciences, University of British Columbia, Vancouver Prostate Centre, Vancouver, Canada; Department of Urologic Sciences, University of British Columbia, Vancouver Prostate Centre, Vancouver, Canada; British Columbia Cancer Research Centre, 675 West 10th Avenue, Vancouver, British Columbia, Canada and Department of Pathology, University of British Columbia, Vancouver, British Columbia, Canada; Department of Urologic Sciences, University of British Columbia, Vancouver Prostate Centre, Vancouver, Canada

## Abstract

We report a new mechanism of androgen receptor (AR) mRNA regulation and cytoprotection in response to AR pathway inhibition (ARPI) stress in prostate cancer (PCA). AR mRNA translation is coordinately regulated by RNA binding proteins, YTHDF3 and G3BP1. Under ambient conditions m6A-modified AR mRNA is bound by YTHDF3 and translationally stimulated, while m6A-unmodified AR mRNA is bound by G3BP1 and translationally repressed. When AR-regulated PCA cell lines are subjected to ARPI stress, m6A-modified AR mRNA is recruited from actively translating polysomes (PSs) to RNA-protein stress granules (SGs), leading to reduced AR mRNA translation. After ARPI stress, m6A-modified AR mRNA liquid–liquid phase separated with YTHDF3, while m6A-unmodified AR mRNA phase separated with G3BP1. Accordingly, these AR mRNA messages form two distinct YTHDF3-enriched or G3BP1-enriched clusters in SGs. ARPI-induced SG formation is cell-protective, which when blocked by YTHDF3 or G3BP1 silencing increases PCA cell death in response to ARPI stress. Interestingly, AR mRNA silencing also delays ARPI stress-induced SG formation, highlighting its supportive role in triggering this stress response. Our results define a new mechanism for stress adaptive cell survival after ARPI stress involving SG-regulated translation of AR mRNA, mediated by m6A RNA modification and their respective regulatory proteins.

## INTRODUCTION

Androgen receptor (AR) pathway inhibition (ARPI) induces profound and sustained responses in advanced prostate cancer (PCA). Unfortunately, recurrence is inevitable and associated with re-activation of the AR and progression to castration-resistant prostate cancer (CRPC) (1,2), attributable to genomic and metabolic re-activation of the AR (3) and supported by context-dependent activation of stress response, kinase signaling, and developmental pathways (4–10). These mechanisms work in concert and highlight the AR as a central oncogenic driver of progression and treatment resistance in PCA. Indeed, ARPI-induced AR gene amplifications and mutations are the most common genomic alterations in CRPC (1,11–13), and ARPI induces expression of both AR-_FL_ and AR-_Variant_ mRNA in preclinical models (14) and human PCA (15). While adaptive responses supporting clonal evolution emphasize transcriptional, epigenetic, and mutational changes, emerging evidence includes stress adaptation through acute changes in mRNA trafficking and translation (16). Moreover, genomic and transcriptomic changes insufficiently predict biology, and mRNA and protein expression levels frequently do not correlate (17–21). Cancer cells can co-opt key homeostatic stress responses, including adaptive changes in mRNA translation that contribute to cell survival and therapy resistance, but little is known about roles for AR mRNA and regulation of its translation during ARPI stress.

A highly conserved mechanism for regulation of translation during stress involves the sequestration and protection of mRNAs in stress granules (SGs). SGs are membrane-less ribonucleoprotein (RNP) complexes formed by liquid-liquid phase-separation (LLPS) of intrinsically disordered proteins and RNAs, comprised of mRNAs, ribosomal components (40S), RNA-binding proteins (RBPs) and signalling factors (22–27). SGs sequester translationally repressed mRNAs, and allow the selective translation of a subset of cytoprotective mRNAs that are excluded from SGs (16,28–30). Previously, we discovered that the highly conserved RNA-binding protein (RBP), YB-1, directly binds to the 5′-UTR of SG nucleator G3BP1 for translational activation, facilitating SG formation in many cancer types under stress; moreover, inactivation of YB-1 or G3BP1 reduced invasive and metastatic capacity (31). Our recent research suggests that during SG formation, G3BP1 guides transcript partitioning to reprogram mRNA translation and support cell protection in PCA (16). These studies imply a functional role for these novel RNA-protein complexes, and that targeting SG formation might be exploitable as a therapeutic strategy.

An important regulator of mRNA homeostasis involves RNA epitranscriptomic modifications (ETMs) (32–34). Methylation (–CH_3_) at the N6-position of adenosine (m6A) is an abundant and versatile RNA ETM (35–39). Writers, readers and erasers coordinately regulate RNA m6A ETMs and various aspects of RNA metabolism, including splicing, nuclear export, localization, translation and stability. Writers are methylases that add m6A to RNA (e.g., METTL3, METTL14, WTAP, KIAA1429), readers are mainly RBPs that bind to m6A-modified RNA (e.g., YTHDF1-3, YTHDC1), and erasers are demethylases that remove m6A from RNA (e.g., FTO, ALKBH5) (37). Disturbances of RNA ETM-regulatory writers, readers and erasers are implicated in several diseases, including obesity and cancer (38,40).

In this study, we investigated AR mRNA regulation in the context of acute ARPI stress to define rapid adaptive changes that support survival under treatment stress. Depending on its m6A status, YTHDF3 and G3BP1 RBPs coordinately regulated the translation of AR mRNA. Under ambient conditions, YTHDF3 binds an m6A-modified, translationally active fraction of AR mRNA in polysomes (PSs), while G3BP1 binds an m6A-unmodified, translationally inactive fraction of AR mRNA. When AR-regulated PCA cells are exposed to ARPI stress, AR messages traffic away from PSs, and translation of AR mRNA is reduced. Translationally-repressed AR mRNAs assemble in the cytosol in SGs to associate in distinct clusters with YTHDF3 and G3BP1. Blocking this adaptive response by silencing of YTHDF3 or G3BP1 sensitizes PCA cells to ARPI stress. Interestingly, AR (or ESR1) silencing also blocked SG formation after ARPI (or ERPI) stress in prostate (and breast) cancer cells, highlighting a role for AR and ESR1 mRNA in triggering SGs when its receptor is antagonised. Our research defines a novel mechanism of AR mRNA translation mediated by m6A modification, its regulatory RBPs, and SG formation.

## MATERIALS AND METHODS

### Cell lines, antibodies and reagents

LNCaP, V16-D and PC-3 cells were obtained from ATCC. The following primary antibodies were used: PARP (Cat. No. 9542), BAX (2D2) (Cat. No. 89477), β-Actin (Cat. No. 3700), BIOTIN (Cat. No. 5597), AR (Cat. No. 5153) and GAPDH (Cat. No. 2118) were from Cell Signaling; G3BP1 (Cat. No. 611127) was from BD Biosciences; YB-1 (Cat. No. ab76149), m6A (Cat No. ab151230) and G3BP1 (Cat. No. ab56574) were from Abcam; YTHDF3 (Cat. No. NBP2-94636) was from Novus; m6A (Cat. No. 202 003) was from Synaptic Systems; ERα (Cat. No. SAB4500810) and Vinculin (Cat. No. V9131) were from Sigma; Fluorescent secondary antibodies (mouse, Alexa Fluor 488/594; rabbit, Alexa Fluor 488/594), TRIzol, RPMI, FBS, RNAiMAX transfection reagent, Dynabeads M-280 Streptavidin, DMEM, FBS, Click-iT Protein Reaction Buffer Kit, biotin-alkyne and L-azidohomoalanine (AHA) were from Life Technologies; FluorSave was from Merck; siAR siRNA (5′-UCAAGGAACUCGAUCGUAUUU-3′) targeting full-length AR was from Dharmacon; siESR1 (Cat. No. sc-29305), siMETTL3 (Cat. No. sc-92172), siWTAP (Cat. No. sc-63224), siKIAA1429 (Cat. No. sc-77700), siMETTL14 (Cat. No. sc-89054), and siControl (Cat. No. sc-37007) siRNAs were from Santa Cruz Biotechnology.

### Immunoblotting

Untreated or ARPI stressed LNCaP cells were gently scraped off from the culture dishes with a cell scraper, washed with PBS, and lysed using lysis buffer (20 mM Tris–HCl, pH 7.5, 150 mM NaCl, 1 mM Na_2_EDTA, 1 mM EGTA, 1% Triton X-100 and 1× protease inhibitor). Cell lysates were centrifuged at 5000 rpm for 10 min and the supernatant was saved. Protein concentration was determined using a Bradford assay (Bio-Rad Laboratories). Protein lysates were mixed with 2× loading dye, and equal amount of proteins were separated in 4–12% gradient SDS-PAGE and immunoblotted into nitrocellulose membrane using wet transfer as described previously (41). Newly synthesized proteins were analysed using click chemistry (42). Briefly, LNCaP cells were incubated with azidohomoalanine (AHA) for 1 h to incorporate the AHA into newly synthesized proteins. The AHA labeled proteins lysates were subjected to Click reactions for 1 h with 40 μM biotin-alkyne using Click-iT protein reaction buffer kit as per the manufacturer's instructions (Life Technologies). Total proteins from Click reactions were precipitated with methanol/chloroform and resolubilized in 50 mM Tris, pH 7.5, 0.01% SDS in a concentration of 1 μg/μl. Biotin-tagged proteins (1 mg) were then incubated with 50 μl of Streptavidin coupled magnetic beads (Dynabeads M-280 Streptavidin, Invitrogen) for 5 h at room temperature. After extensive washing in PBS and 0.5% SDS × 5 times (20 min each) to remove nonspecific binding or protein–protein interactions, resin suspensions were incubated in 50 μl of 2× loading dye for 10 min at 95°C to separate out the tagged proteins from beads. The immunoprecipitated proteins were subjected to immunoblotting.

### Immunofluorescence (IF) and immunohistochemistry (IHC)

Cells seeded at 20–25% confluence in 6-cm culture dishes containing round cover glasses (12CIR-1D; Thermo Fisher Scientific) were treated with vehicle alone or exposed to ARPI stress. IF was performed as described previously (31). Cells were fixed in 4% paraformaldehyde (PAF) for 20 min and permeabilized with PBS-T (0.05% Triton X-100 in PBS) for 20 min. The cells were then blocked for 30 min in PBS-T containing 5% BSA and incubated with primary antibodies (1:100) for 1 h in PBS containing 2.5% BSA. Cells were washed in PBS-T for 30 min (3 × 10 min) followed by incubation with secondary antibodies (1:200) in PBS-T containing 2.5% BSA for 1 h. Cells were then washed in PBS-T for 30 min (3 × 10 min). In the case FFPE tissues, a Roche Ventana BenchMark ULTRA Slide Stainer system was used for deparaffinization and activation of antigen epitopes, and IF staining was carried out as described above. The IF slides were immersed in DAPI (10 μM) for nuclear staining, mounted with FluorSave, and viewed using Zeiss LSM780, confocal microscope with 60× and 100× oil-immersion objective lenses. Images were captured using ZEN imaging software. To identify G3BP1 and YTHDF3 clusters in unstressed and stressed cells, the immunostained slides were viewed using Leica SP8 X STED White Light Laser Confocal Microscope with 100× oil-immersion and the images were processed using Huygens Professional Deconvolution Software Package (Histology Core Lab, BC Children's Hospital). To detect the co-localization of m6A-modified mRNAs with G3BP1 or YTHDF3, proximity ligation assay was performed using a commercially available Duolink PLA kit (Cat. No. DUO92101 from Sigma-Aldrich). For IHC of patient tumors, different human PCA tissue microarrays (Pathology Core, VPC) were subjected to IHC using anti–G3BP1 and anti-YTHDF3 antibodies. Sections were counterstained with hematoxylin and viewed using a light microscope (Zeiss Axio Observer Z1) with 10× and 40× objective lenses. Images were captured using ZEN software. A board-certified pathologist scored the staining for each antibody. A Turkey's multiple comparison analysis was performed to assess the expressions of each protein.

### In situ hybridization (ISH)

For the localization of AR mRNA, manual in situ hybridization (ISH) was performed as described previously (16). Probes for AR (Cat. No. 400491) and non-specific negative control targeting the DapB gene from the Bacillus subtilis strain SMY (Cat. No. 310043) were purchased from Advanced Cell Diagnostics (ACD). For sample preparation, we followed ‘Sample Preparation Technical Note for Cultured Adherent Cells Using RNAscope^®^ 2.5 Chromogenic Assay (Single-plex and Duplex)’ from ACD. Briefly, LNCaP cells seeded in glass-bottom chamber slides were transfected with siControl or siAR siRNAs for 48 h. Cells were then subjected to +/–ARPI stress and fixed in 4% PAF. Cells were dehydrated in a Coplin jar using 50%, 70% and 100% ethanol, then rehydrated in 70%, 50% ethanol and 1× PBS (each for 10 min). Slides were treated with hydrogen peroxide, quick protease III to digest proteins to enhance probe accessibility to RNA, and then subjected to hybridisation using RNAscope^®^ 2-Plex Detection Kit (Chromogenic). First, slides were hybridised with a mRNA specific probe for 2 h, then incubated with a series of probes (Amp 1–6) provided by ACD to enhance the hybridization signal, followed by treatment with Fast A substrate to reveal red spots corresponding to the regions of mRNA localisation. The ISH slides were then subjected to immunostaining with anti-G3BP1 or ant-YTHDF3 antibodies as described above and viewed using Zeiss LSM780, confocal microscope with 60× and 100× oil-immersion objective lenses. Images were captured using ZEN imaging software.

### Isolation of total RNA, polysomal RNA, riboimmunoprecipitation (RIP) and qRT-PCR

Total RNA was extracted using TRIzol reagent according to the manufacturer's instructions. Polysomal mRNA was extracted using a protocol described before (16). For the extraction of mRNAs associated with YTHDF3 in the PSs, PSs were extracted from unstressed and ARPI stressed cells, YTHDF3 was pulldown from the polysome fractions using anti-YTHDF3 antibodies and the YTHDF3 associated mRNAs were extracted using TRIzol (31). For the purification of m6A-modified mRNAs in the PSs, mRNAs were extracted from PSs of unstressed and ARPI stressed cells using TRIzol and these mRNAs were subjected pulldown using anti-m6A antibodies (36). The amount of RNA from the above preparations were quantified using NanoDrop (Thermo Fisher Scientific). cDNAs were synthesized using the QuantiTect Reverse Transcription kit. Equal amounts of cDNAs were subjected to qRT-PCR in an ABI PRISM 7500 Sequence Detection System (Applied Biosystems) with PCR conditions 50° for 2 min, 95°C for 10 min, 95°C for 15 s (40 cycles) and 60°C for 1 min (40 cycles). See Supplementary Table S1 for primers used for qRT-PCR.

### Purification of recombinant proteins

G3BP1_OHu02150C_pET-28a(+)-TEV, YTHDF3_OHu08859C_pET-28a(+)-TEV, YTHDF3-EGFP_YTHDF3_OHu08859C_pET-28a(+)-TEV, G3BP1-RFP_G3BP1_OHu02150C_pET-28a(+)-TEV were custom cloned from GenScript. The plasmids were transfected to BL21(DE3) pLysS competent cells (Promega). Positive colonies were picked up and grown at 37°C in LB media supplemented with 50 μg/ml of kanamycin until the early exponential phase (OD600 0.4–0.8). Isopropyl-β-d-1-thiogalactopyranoside (IPTG) was added to the culture to a final concentration of 0.5 mM and protein expression was induced by further incubating the culture at 37°C for 5 h. The cells were harvested by centrifugation at 7000 rpm for 10 min, resuspended in 10 ml lysis buffer (10 mM imidazole in 1× PBS) and subjected to sonication. The suspension was then cleared by centrifugation at 10 000 rpm for 30 min at 4°C and the resulting supernatant was passed through a 1 ml Ni-NTA column (Thermo Scientific). After washing the column with washing buffer (50 mM imidazole in PBS), recombinant proteins were eluted with elution buffer (250 mM imidazole in PBS) and dialyzed extensively against PBS to remove the imidazole.

### RNA electrophoretic mobility shift assay (REMSA)

RNA EMSA to detect direct binding of G3BP1 or YTHDF3 to m6A-unmodified or m6A-modified probes was performed using a LightShift Chemiluminescent RNA EMSA kit (Thermo Fisher Scientific) according to the manufacturer's instructions. In brief, 6.25 nM biotin tagged probes (see Supplementary Table S1 for probe sequences) were incubated with recombinant G3BP1 or YTHDF3 in binding buffer (100 mM HEPES, pH 7.3, 200 mM KCl, 10 mM MgCl_2_, 10 mM DTT, 5% glycerol and 2 μg tRNA) in a total volume of 20 μl for 30 min. Control reactions were set up using 200-fold molar excess of unlabeled RNA probe added together with labeled RNA. The reaction products were mixed with 5 μl of 5× loading buffer and subjected to electrophoresis in a 6% native polyacrylamide gel in 0.5× TBE buffer (100 V for 8 × 8 × 0.1 cm gel) until the bromophenol blue dye has migrated 3/4 down the length of the gel. The RNA–protein complexes were then transferred to a nylon membrane and cross-linked for 60 s at 120 mJ/cm^2^ using a commercial UV light cross-linker (Agilent Technologies) equipped with 254-nm UV light lamps. The membranes were then incubated with stabilized Streptavidin-Horseradish peroxidase conjugate and developed using chemiluminescent substrate.

### RNA affinity chromatography

The RNA affinity chromatography was performed as described previously (31). LNCaP cells were harvested and homogenized in binding buffer containing 10 mM Tris–HCl, pH 7.4, 1.5 mM MgCl_2_, 10 mM KCl, 0.05% NP-40 and 1× protease inhibitors using a Dounce homogenizer. The lysates were centrifuged at 10 000 g at 4°C for 10 min, and the supernatant was saved. The lysates (2 mg) were then precleared by incubating with 20 μl of RNasin (RNase inhibitor), 12 μg of yeast tRNA, and 100 μl Streptavidin magnetic beads in a total volume of 1 ml at 4°C for 2 h. In parallel, 100 μg of biotinylated RNA was incubated with 100 μl of Streptavidin magnetic beads at 4°C for 2 h on a rotator. The beads were washed and mixed with the precleared lysate and incubated at 4°C for 30 min in a rotator, then washed five times in the binding buffer, boiled in loading dye, and subjected to immunoblotting.

### Phase-separation assays

Phase-separation assays were conducted as described previously with modifications (43,44). Briefly, LLPS of G3BP1 or YTHDF3 was induced by adding crowding agent Ficoll 400 or RNA. To test the effect of crowding agent on phase separation, recombinant G3BP1 or YTHDF3 in 1× PBS (20 mM sodium phosphate, 300 mM NaCl, pH 7.4) at a concentration of 5 μg/μl was mixed with Ficoll 400 solution to achieve a final concentration of 50 μM of protein and Ficoll 400 (0%, 2%, 4%, 8% and 16%) in a microfuge tube and incubated for 2–5 min at room temperature. The phase-separated suspension (5 μl) was pipetted into a glass-bottom dish and imaged under a phase-contrast microscope (Zeiss Axio Observer Z1) under 20× and 32× objectives. To determine the surface wetting of G3BP1 and YTHDF3 droplets, the phase-separated droplets were either incubated in the microfuge tube or pipetted onto a glass-bottom dish and incubated for different time points (5, 10, 15 and 20 min) at room temperature and imaged as above. To test the effect of salt and pH on phase separation G3BP1 and YTHDF3, total RNA (20 ng/μl) was mixed with recombinant G3BP1 or YTHDF3 in 20 mM sodium phosphate containing 25, 50, 100 and 200 mM NaCl at pH 4, 6 and 7, and imaged as above. To check the effect of temperature on the phase-separated droplets, G3BP1 or YTHDF3 droplets (in phosphate buffer, pH 7.4, containing 25, 50 or 100 mM NaCl) were either kept at room temperature or at 37°C before imaging as described above. For invitro G3BP1 and YTHDF3 clustering assays, phase separation was induced in a microfuge tube by the addition of total RNA (20 ng/μl) to RFP-G3BP1 (50 μM) or GFP-YTHDF3 (62 μM), the different droplets were mixed, immediately pipetted to a glass-bottom dish and imaged using confocal microscopy. To study phase-separation of AR mRNA in G3BP1 or YTHDF3 droplets, total RNA was added to G3BP1 or YTHDF3 to induce phase-separation in a total volume of 250 μl in a microfuge tube. The phase-separated droplets were then centrifuged (250 rpm for 5 min) to separate two fractions, the bottom ‘droplet’ and the upper ‘liquid’ fractions. The upper ‘liquid’ fraction was carefully pipetted into a fresh tube, leaving the bottom ‘droplet’ fraction in the same tube. Aliquots (5 μl) from each fraction were pipetted into a glass-bottom dish and imaged to assess the quality of the separated fractions. RNAs associated with each fraction were extracted using TRIzol and subjected to qRT-PCR using AR specific primers. To identify m6A-modified AR mRNAs, m6A-modified mRNAs were pulldown using EpiMark^®^ N6-methyladenosine Enrichment Kit (NEB) from the supernatant and droplets fractions and the pulldown mRNAs from each fraction were subjected to qRT-PCR using AR specific primers. To check the effect of requirement of RNA in the phase-separation, recombinant G3BP1 or YTHDF3 were mixed with RNA in presence and absence of RNase A and subjected to phase separation and imaged as explained above.

### AR transactivation assay

LNCaP cells were transfected with siRNA for either G3BP1, YTHDF3, METTL3 or scrambled control at the time of seeding in 96-well plate using Lipofectamine RNAimax as per manufacturer's guidelines. Next day, the cells were transfected with 50 ng of reporter plasmid ARR3tk-luciferase using TransIT2020 transfection reagent (Mirus, Madison, WI, USA). After 24 h of transfection, the cells were untreated or treated with ARPI stress. Luciferase activity was measured using the dual luciferase assay system (Promega, Madison, WI) and normalized by Renilla activity or protein concentration (45).

### Measurement of cellular proliferation

LNCaP cells were transfected with siControl, siG3BP1 or siYTHDF3 siRNAs using Lipofectamine RNAimax. The transfected cells were treated with 10 μM ENZA. After 24 h of ENZA treatment, cells were treated with 20 μM ARS. For rescue experiment, media in treated (ENZA+ARS) cells were replaced with fresh media without compounds. All cells were incubated in a IncuCyte^®^ S3 Live-Cell Analysis System for real time image acquisition. Cell proliferation data was quantified using the IncuCyte^®^ basic analyzer.

### Statistics

Statistical analyses were performed in GraphPad Prism 8 or Microsoft Excel using one-way ANOVA and two-tailed unpaired Student's t-test unless otherwise indicated. The outcomes of all statistical tests including p-values and number of experiments are included in the figure legends. Significance was defined as any statistical outcome that resulted in a *P* < 0.05, unless otherwise indicated. P- value significance is represented by the following: *P < 0.05, **P < 0.01, ***P < 0.001, ****P < 0.0001.

## RESULTS

### PCA cells and tumor tissues form SGs in response to ARPI stress

**Figure 1.**
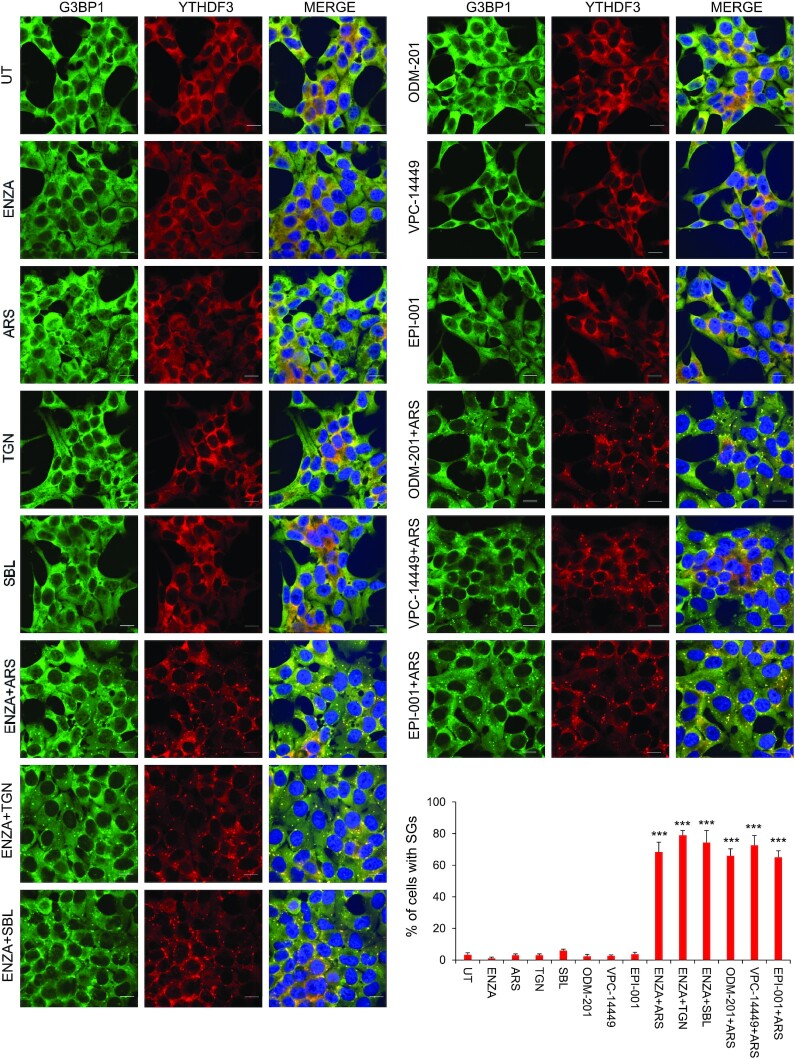
ARPI stress induces SG formation in LNCaP cells. LNCaP cells were unstressed or pre-treated with enzalutamide (ENZA) for 24 h, and followed by treatment with arsenite (ARS), thapsigargin (TGN) or sorbitol (SBL). The cells were immunostained with anti-G3BP1 and anti-YTHDF3 antibodies to reveal the SGs (left-side panel). LNCaP cells were pre-treated with ARPI drugs ODM-201 (Darolutamide), VPC-14449 or EPI-001 for 24 h and followed by treatment with ARS for 1 h. The cells were immunostained with anti-G3BP1 and anti-YTHDF3 antibodies to reveal the SGs (right-side upper panel). Quantification of SGs is shown in the right-side bottom panel. Note that ARPI stress induces SGs, while ARPI or individual stresses alone do not induce SGs. The results are an average of three independent experiments with ***P < 0.001. Scale 10 μm.

**Figure 2.**
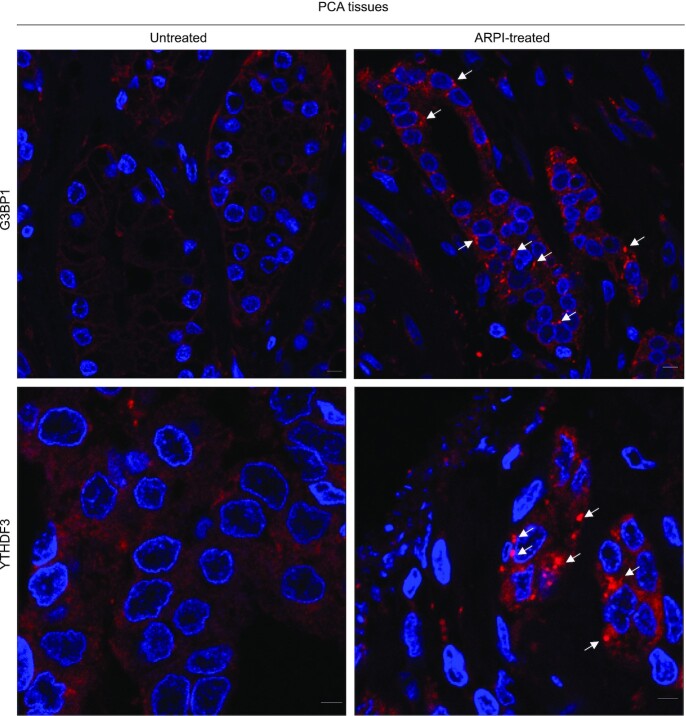
ARPI treatment induces SGs in PCA tissues. PCA tumor samples from untreated or ARPI-treated patients were immunostained with anti-G3BP1 or anti-YTHDF3 antibodies. Note that SGs (indicated by white arrowheads) were formed in tissues from patients exposed to ARPI-treatment. Scale 10 μm.

Since ARPI is stressful for AR-responsive PCA cells, we explored conditions whereby this treatment induced SG formation. When LNCaP cells were pre-treated with the AR-antagonist enzalutamide (ENZA), SGs efficiently formed when combined with a mild stress, including oxidative stress [arsenite (ARS) 20 μM for 1 h], endoplasmic reticulum (ER) stress [thapsigargin (TGN) 0.1 μM for 1 h], or osmotic stress [sorbitol (SBL) 50 μM for 1 h]. SGs were visualized by co-localization staining of SG marker proteins G3BP1 and YTHDF3 (Figure 1) or G3BP1 and YB-1 (Supplementary Figure S1). We defined this combined stress, i.e. treatment with mild cellular stress plus ARPI as ‘ARPI stress’, which aims to model anti-cancer treatment in a stressed tumor microenvironment (46–48). Mild stress alone, or ARPI alone, did not induce SGs (Figure 1 and Supplementary Figure S1). We also observed enhanced SG formation with other AR-antagonists, including darolutamide (ODM-201), VPC-14449 and EPI-001 (49–51), co-treated with mild oxidative stress (Figure 1 and Supplementary Figure S1). We tested an additional AR-sensitive PCA cell line V-16-D, and found similar induction of SGs in response to ARPI stress (Supplementary Figure S2A). AR negative PC-3 cells were insensitive to ARPI stress and did not form SGs (Supplementary Figure S2B), suggesting that an active AR pathway is necessary to form SGs in ARPI-stressed cells. The AR antagonist bicalutamide also induced SGs similar to enzalutamide but interestingly, other therapeutics approved in advanced PCA, including the microtubule inhibitor docetaxel and the PARP inhibitor olaparib, did not induce SG formation when combined with a low dose of arsenite and used at concentrations that reduced cell survival (Supplementary Figure S3A and B). SGs also form in clinical PCA tissues from patients treated with ARPI, with more SGs apparent after neo-adjuvant ARPI compared to treatment-naïve cancers (Figure 2). Collectively, these results suggest an AR-centric context-dependent induction of SGs in AR^+^ PCA cell lines and clinical PCA tumors after ARPI stress.

### AR mRNA transcripts are recruited to SGs after ARPI stress

**Figure 3.**
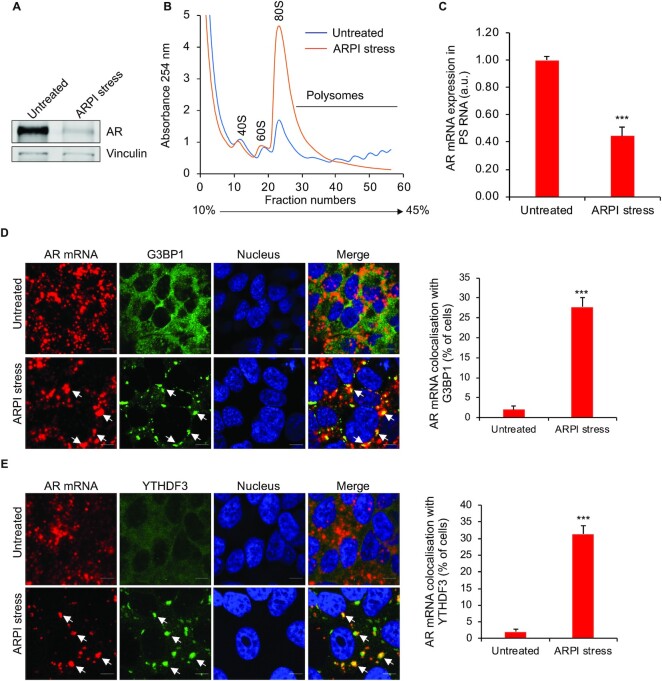
ARPI stress reduces the expression of AR protein. (**A**) Western blot showing reduced AR protein expression after ARPI stress. (**B**) The PS trace of untreated or ARPI-stressed cells. (**C**) qRT-PCR showing ARPI stress-induced reduction in AR mRNA in the pooled PS fractions. (D and E) In situ hybridization (ISH) for AR mRNA in unstressed and ARPI stressed cells. Cells were counterstained with G3BP1 (**D**) or YTHDF3 (**E**). Arrowheads indicate the co-localisation of AR mRNA with G3BP1 or YTHDF3. Quantification of co-localization of AR mRNA with G3BP1 or YTHDF3 is shown on the right-side panels. The results are an average of three independent experiments with ***P < 0.001. Scale 10 μm.

AR protein levels have been reported to decrease after ARPI (52), which we confirmed in ARPI stressed cells (Figure 3A). Total transcript levels of AR (Supplementary Figure S4A) or degradation rates of AR protein (Supplementary Figure S4B) are not affected by ARPI stress, excluding ARPI stress effect on AR mRNA transcription or AR protein degradation. However, by sucrose gradient polysome (PS) fractionation, we found a significant reduction of AR mRNA in PSs of ARPI-stressed cells compared to unstressed cells (Figure 3B and C), indicating that reduced AR protein levels correlated with its reduced mRNA association with PSs. By monitoring the relative distribution of AR mRNA across the sucrose gradient, we found that the AR mRNA is redistributed from the translatable PS fractions in untreated cells to untranslatable non-PS fractions in ARPI stressed cells (Supplementary Figure S5). Since SGs can phase separate translationally stalled mRNAs in response to stress (22), we tested whether the stalled AR mRNA from the PSs is recruited to SGs after ARPI stress. In situ hybridization (ISH) using oligos corresponding to AR messages revealed its co-localization with both G3BP1 and YTHDF3 in SGs after ARPI stress (Figure 3D and E). No hybridisation signal was detected in AR knockdown (KD) cells (Supplementary Figure S6A) or with a non-specific negative control probe targeting the DapB gene (Bacillus subtilis strain SMY, a soil bacterium), indicating the specificity of the ISH (Supplementary Figure S6B). Together, these results suggest that acute ARPI stress induces disassembly of AR mRNA from PSs and recruitment to SGs and reduced AR protein synthesis.

### YTHDF3 binds m6A-modified, and G3BP1 binds m6A-unmodified, AR mRNA

**Figure 4.**
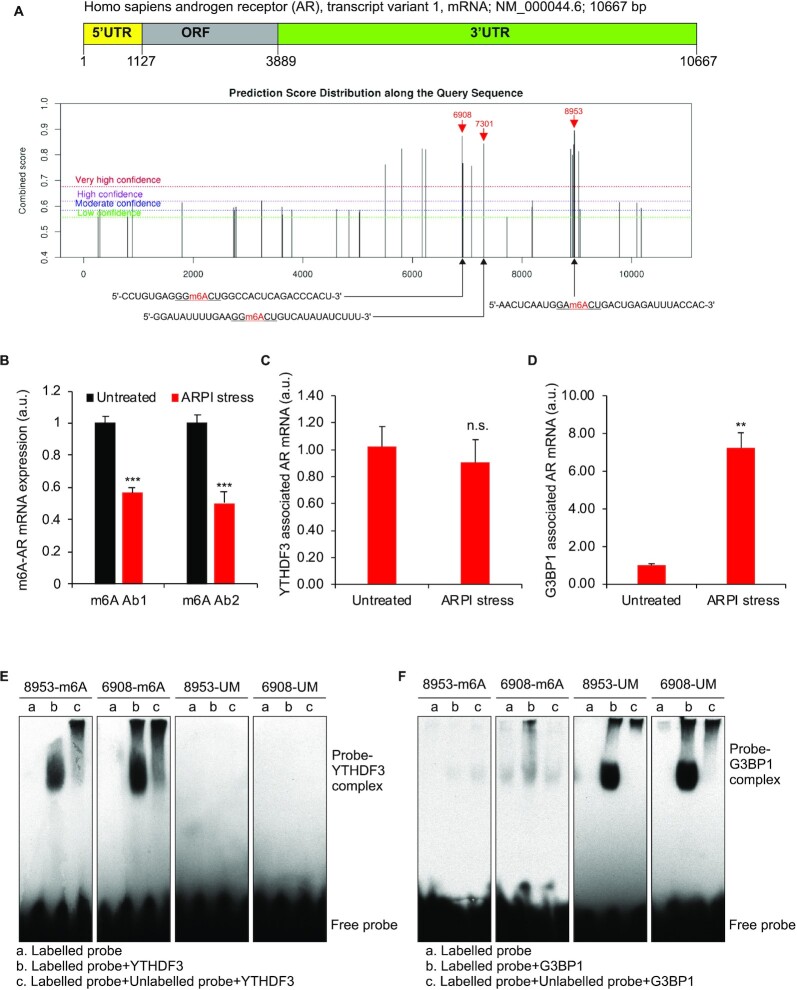
AR mRNA is m6A-modified and differentially binds YTHDF3 and G3BP1. (**A**) Prediction of m6A modifications in AR mRNA using SRAMP m6A prediction software (http://www.cuilab.cn/sramp/). Three m6A modification regions (positions: 8953, 7301, and 6908) with very high confidence, and RNA probes corresponding to those regions are indicated. (**B**) qRT-PCR for m6A-modified AR mRNA in total RNA pulldown from unstressed and ARPI stressed cells using two different anti-m6A antibodies (m6A-Ab1 and m6A-Ab2). (C, D) Interaction of AR mRNA with YTHDF3 (**C**) and G3BP1 (**D**) in unstressed and ARPI stressed cells. The results are an average of three independent experiments with ***P < 0.001, **P < 0.01. n.s., non-significant. (E, F) RNA EMSA to analyse the direct binding of YTHDF3 or G3BP1 to m6A-modified or unmodified probes corresponding to regions 8953 and 6908 of AR mRNA. Biotin-labeled probes were mixed with recombinant YTHDF3 (**E**) or G3BP1(**F**) and subjected to EMSA. A probe mobility shift in the presence of YTHDF3 or G3BP1 and free probe are indicated. A 200-fold molar excess concentration of unlabeled probe was added along with the labelled probe to demonstrate the specificity of RNA–protein complex formation (see Results section for more details).

m6A-modified mRNA has been reported to bind YTHDF3 and not G3BP1 (53). Since AR mRNA transcripts co-exist with YTHDF3 or G3BP1 in SGs in ARPI stressed cell (Figure 3D and E), we set out to define effects of m6A modification on AR mRNA interactions with these RNA binding proteins (RBPs). In silico m6A prediction (54) identified m6A-modified residues in AR mRNA (Figure 4A). Global m6A sequencing studies also catalogued AR as an m6A-modified mRNA (39). To determine the m6A-modification of AR mRNA, m6A-modified messages were pulled down from total RNA extracted from unstressed or ARPI-stressed LNCaP cells using two different anti-m6A antibodies (m6A Ab1, Cat No. ab151230, Abcam; m6A Ab2, Cat. No. 202 003, Synaptic Systems) and quantified by qRT-PCR for AR mRNA. Figure 4B illustrates that AR mRNA is m6A-modified under basal condition and that levels of m6A-modified AR mRNA decrease after ARPI stress.

To determine the association of AR mRNA with YTHDF3 and G3BP1 in cells, both proteins were pulled down from untreated or ARPI-stressed LNCaP cells using ribo-immunoprecipitation (ribo-IP) and the associated AR mRNA was analyzed by qRT-PCR. We found that both G3BP1 and YTHDF3 binds AR mRNA in untreated cells. While AR mRNA-YTHDF3 interaction remained unchanged following stress, the AR mRNA-G3BP1 interaction increased after stress (Figure 4C and D). To characterize the direct interactions of AR mRNA with YTHDF3 and G3BP1, we cloned the ORF (open reading frame) of YTHDF3 and G3BP1, fused with His-tag, expressed and then purified the recombinant proteins (Supplementary Figure S7A and B). The binding of recombinant YTHDF3 or G3BP1 to RNA probes corresponding to m6A-modified or -unmodified AR mRNA was determined using RNA electrophoretic mobility shift assay (EMSA) (31). First, we designed biotin-labelled mRNA probes (30 nucleotides) with or without m6A modifications, corresponding to two different regions of AR mRNA with high m6A prediction scores (Figure 4A). The selected regions contained the two most abundant m6A consensus sequences (55). Probe 8953-m6A with consensus sequence GAm6ACU, and probe 8953-UM with GAACU, for the m6A-modified and -unmodified versions for the position 8953, respectively, while probe 6908-m6A with consensus sequence GGm6ACU and probe 6908-UM with GGACU, for the m6A-modified and unmodified versions for the position 6908, respectively (Figure 4A). Both m6A-modified probes, 8953-m6A and 6908-m6A specifically interacted with YTHDF3, and binding affinity was abolished when an unlabelled competitor probe was used along with the labelled probe, confirming specificity of binding (Figure 4E, left two panels). In contrast, both unmodified probes, 8953-UM and 6908-UM did not bind with YTHDF3 (Figure 4E, right two panels). The binding affinity of G3BP1 to unmodified probes (8953-UM and 6908-UM) was higher (Figure 4F, right two panels) compared to the m6A-modified probes (8953-m6A and 6908-m6A) (Figure 4F, left two panels). The titration of 8953-m6A and 6908-m6A probes with increasing concentrations of recombinant YTHDF3 (Supplementary Figure S8A) and that of 8953-UM and 6908-UM probes with increasing concentrations of recombinant G3BP1 (Supplementary Figure S8B) further confirmed the specificity of RNA probe-protein interaction. Moreover, by using RNA affinity chromatography with biotin end labelled m6A-modified or unmodified probes to pulldown interacting proteins from LNCaP cell lysates, we found that m6A-modified probes enriched YTHDF3 compared to G3BP1, while unmodified probes enriched G3BP1 compared to YTHDF3 (Supplementary Figure S9). These results suggest that YTHDF3 and G3BP1 differentially bind AR mRNA based on its m6A status.

Our predicted potential m6A modification sites in AR mRNA (Figure 4A, 19 regions with a high probability of m6A modification), indicated that positions 8953 and 6908 are likely not the only two m6A modification sites that affect AR mRNA’s binding affinity to YTHDF3 and G3BP1. Therefore, to explore this concept, we selected an additional m6A predicted region (position 7301, which lies between the previous 6908 and 8953 in the AR mRNA sequence; Figure 4A). As described above we have generated biotin-labelled mRNA probes (30 nucleotides) with or without m6A modifications for position 7301 (Probe 7301-m6A with consensus sequence GGm6ACU, and probe 7301-UM with consensus sequence GGACU, for the m6A-modified and -unmodified versions for the position 7301, respectively), and used them in binding studies with recombinant YTHDF3 and G3BP1 using EMSA. Similar to our previous observations, m6A-modified probe corresponding to position 7301 strongly interacted with YTHDF3. In contrast, the unmodified probe did not bind YTHDF3 (Supplementary Figure S10A). The binding affinity of G3BP1 to unmodified probe was higher compared to the m6A-modified probe (Supplementary Figure S10B). The titration of individual probes with increasing concentration of YTHDF3 (Supplementary Figure S10C) and G3BP1 (Supplementary Figure S10D) further confirmed the specificity of RNA probe-protein interaction. These results suggest that although YTHDF3 and G3BP1 differentially bind AR mRNA based on its m6A status, the binding affinity of AR mRNA to G3BP1 or YTHDF3 might not be determined by specific residues; instead, it might be determined by a net effect of multiple m6A’s in the AR mRNA.

### M6A RNA modifications affect phase-separation of AR mRNA with YTHDF3 and G3BP1

**Figure 5.**
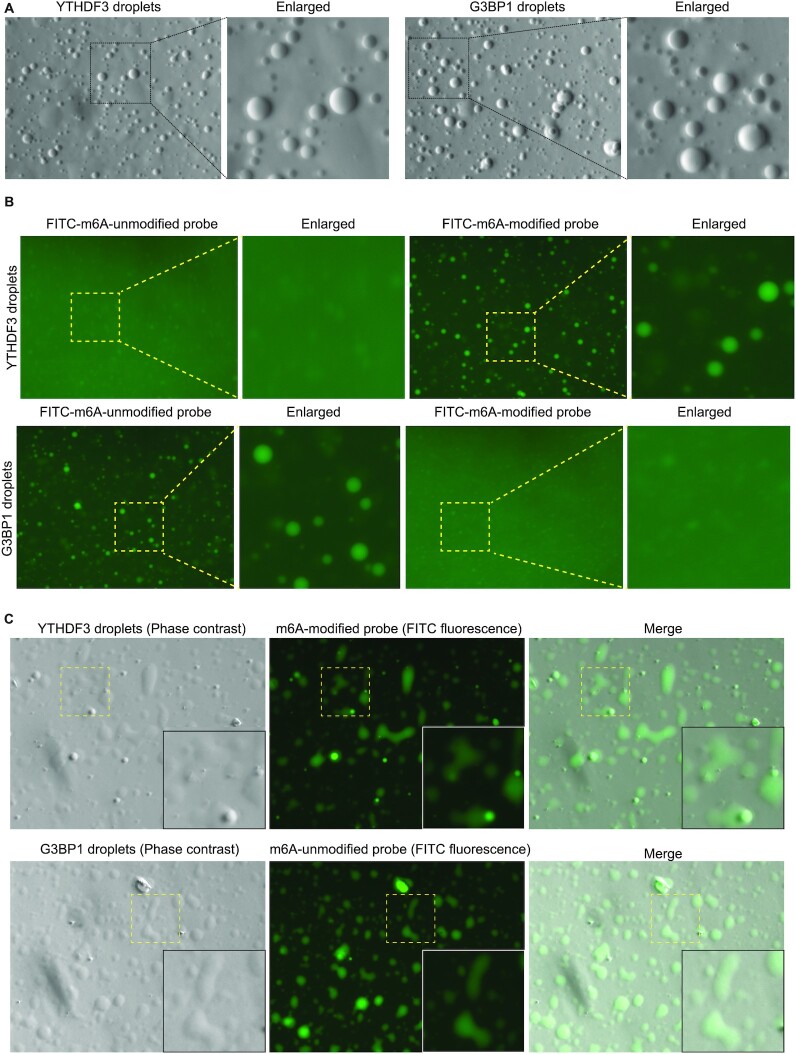
AR mRNA probes phase separated with YTHDF3 and G3BP1. (**A**) LLPS of YTHDF3 and G3BP1 to form liquid droplets. (**B**) m6A-modified FITC-tagged oligos phase separated with YTHDF3, while m6A-unmodified FITC-tagged oligos phase separated with G3BP1. A part of the image is enlarged and shown on the right-side panels. (**C**) YTHDF3 and G3BP1 showed surface wetting, indicating liquid-like property (see Methods for more details). A part of the image is enlarged and shown as an inset.

To investigate effects of m6A modification in recruiting AR mRNA to SGs, we developed an in vitro liquid-liquid phase-separation (LLPS) assay using recombinant YTHDF3 or G3BP1 that formed in vitro liquid droplets (Figure 5A; Supplementary Figure S11) (see Methods for more details). LLPS is a well-established in vitro system to study SG dynamics (44,56–60). FITC-tagged AR mRNA probes (m6A-modified and unmodified; based on position 8953 of AR mRNA) were titrated with YTHDF3 or G3BP1 and subjected to LLPS. m6A-modified AR mRNA probe (8953-m6A) readily phase-separated into YTHDF3 droplets as evidenced by formation of strong green fluorescent droplets, suggesting that m6A-modified AR mRNA oligos can phase separate in YTHDF3 droplets. In contrast, unmodified oligos (8953-UM) showed weak phase-separation with YTHDF3 droplets (Figure 5B). On the other hand, G3BP1 droplets strongly phase separated with unmodified oligos, and only weakly with m6A-modified oligos (Figure 5B). With time the droplets formed by YTHDF3 and G3BP1 wetted the surfaces (Figure 5C), confirming that these droplets had liquid-like properties (59). These results suggest that YTHDF3 and G3BP1 phase separate AR mRNA oligos based on their m6A status.

**Figure 6.**
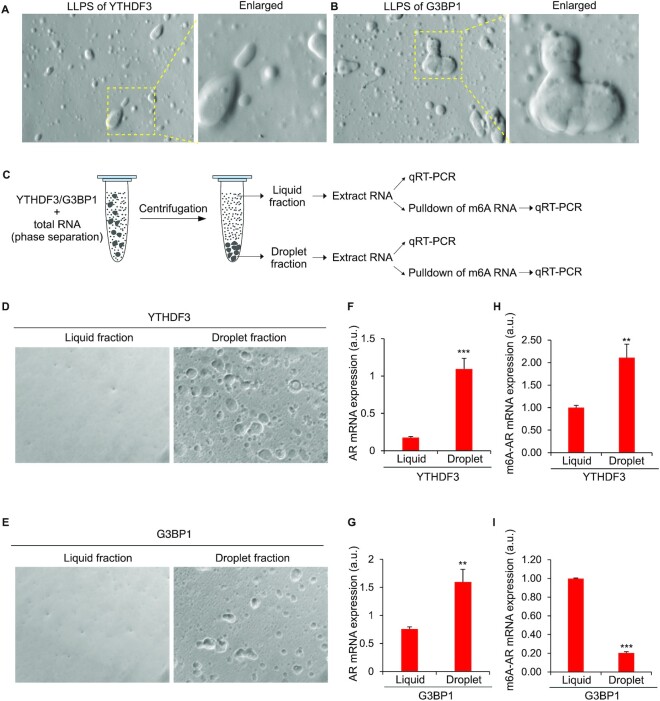
AR mRNA phase separated with YTHDF3 and G3BP1. Total RNA isolated from LNCaP cells were mixed with recombinant YTHDF3 (**A**) or G3BP1 (**B**) to induce liquid–liquid phase-separation (LLPS), and imaged under 32x objective in a light microscope. A part of the image is enlarged and shown on the right-side panels. (**C**) Scheme showing the separation of ‘liquid’ and ‘droplet’ fractions from phase separated YTHDF3 and G3BP1 (see Materials and Methods for more details). Microscopic examination of ‘liquid’ and ‘droplet’ fractions separated by centrifugation of phase separated YTHDF3 (**D**) and G3BP1 (**E**). AR mRNA is more associated with ‘droplet’ fraction compared to ‘liquid’ fraction in YTHDF3 (**F**) and G3BP1 (**G**) phase-separation. (**H**) m6A-modified AR mRNA is more associated with ‘droplet’ fraction compared to ‘liquid’ fraction in YTHDF3 phase-separation. (**I**) m6A-modified AR mRNA is more associated with ‘liquid’ fraction compared to ‘droplet’ fraction in G3BP1 phase-separation. The results are an average of three independent experiments with ***P < 0.001, **P < 0.01.

We next analyzed phase-separation of full-length AR mRNA in the YTHDF3 and G3BP1 droplets. In vitro mixing of YTHDF3 or G3BP1 recombinant protein with total RNA extracted from LNCaP cells induced LLPS to form liquid droplets (Figure 6A and B) as described before (43,44,56). The droplets formed by YTHDF3 and G3BP1 were dynamic and exhibited fission and fusion properties (Supplementary Figure S12; Movie 1 and Movie 2). Both types of droplets were sensitive to alterations in salt (NaCl) concentrations, pH (Supplementary Figure S13) and temperature (Supplementary Figure S14). Droplet formation was not observed or less efficient at a lower pH (pH 4) and salt (NaCl, 25 mM), while it was enhanced at neutral pH (pH 7) and higher salt concentration (NaCl, 200 mM) (Supplementary Figure S13). Although both YTHDF3 and G3BP1 formed droplets at room temperature (23°C) the efficiency of droplet formation was enhanced at a higher temperature (37°C) (Supplementary Figure S14). YTHDF3 or G3BP1, mixed with RNA, did not develop the droplets when the mixtures were RNase A treated, confirming the requirement of RNA in LLPS and droplet formation by YTHDF3 or G3BP1 (Supplementary Figure S15). To determine if AR mRNA is enriched in the above RNA-induced YTHDF3 or G3BP1 droplets, phase-separated fractions were briefly centrifuged (250 RPM for 5 min) to separate the ‘droplet’ fraction from the surrounding ‘liquid’ fraction (see scheme in Figure 6C). When RNA in the ‘droplet’ and ‘liquid’ fractions (Figure 6D for YTHDF3 and Figure 6E for G3BP1) were separately extracted with TRIzol and subjected to qRT-PCR, we found higher levels of AR mRNA in the YTHDF3 and G3BP1 ‘droplet’ fractions compared to surrounding ‘liquid’ fractions, indicating enrichment of AR mRNA in YTHDF3 (Figure 6F) or G3BP1 (Figure 6G) droplets.

Next, to define differences in m6A-modified or -unmodified AR mRNAs phase-separated in YTHDF3 or G3BP1 droplets, m6A -modified messages from ‘droplet’ and ‘liquid’ fractions of YTHDF3 or G3BP1 were pulled down using anti-m6A antibodies, and subjected qRT-PCR to quantify AR mRNA levels (see scheme in Figure 6C). m6A-modified AR messages were enriched in YTHDF3 ‘droplet’ compared to ‘liquid’ fractions, indicating that YTHDF3 droplets mainly phase-separated m6A-modified AR messages (Figure 6H). In contrast, with G3BP1, m6A-modified AR messages were enriched in the ‘liquid’ compared to ‘droplet’ fractions, indicating that AR mRNAs phase-separated in G3BP1 droplets were mainly m6A-unmodified messages (Figure 6I). These experiments revealed that YTHDF3 droplets have a higher affinity to phase separate m6A-modified AR messages, while m6A-unmodified AR messages phase separated with G3BP1.

### AR mRNA supports SG formation in ARPI stressed cells

**Figure 7.**
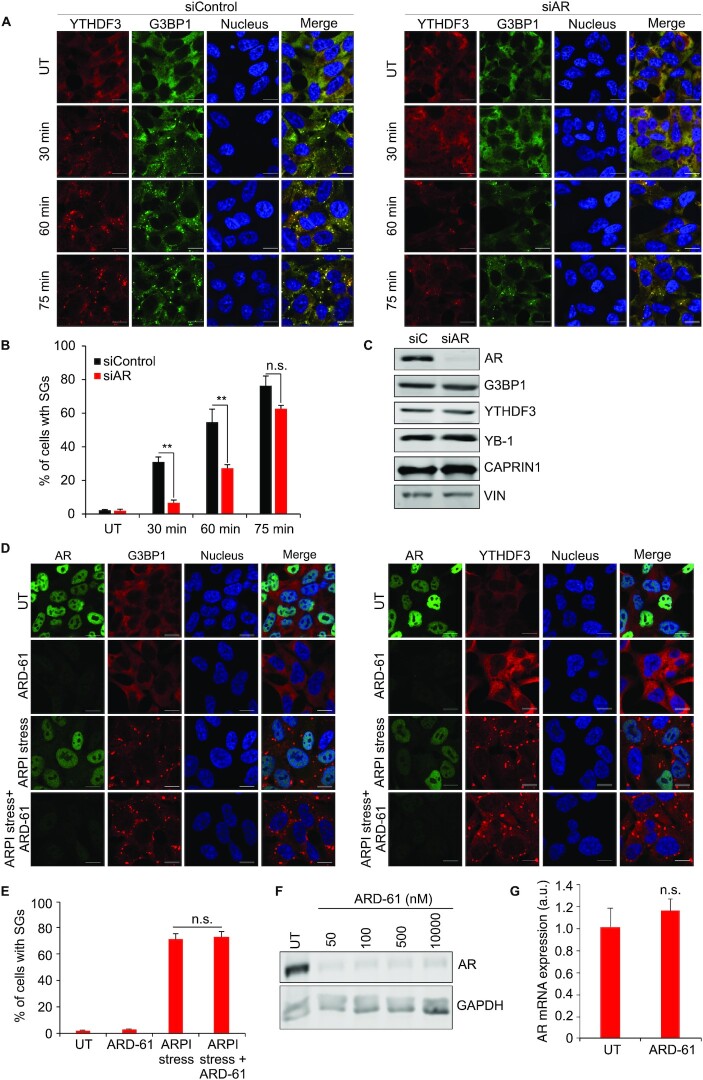
AR mRNA supports SG formation in response to ARPI stress treatment. (**A**) AR knockdown (KD) using siRNAs delayed the formation of SGs. LNCaP cells were transfected with siControl or siAR siRNAs, -/+ ARPI stress for different time points (30 min, 60 min and 75 min), and stained with anti-YTHDF3 and anti-G3BP1 antibodies to analyse SGs. Note that SG formation is significantly delayed at 30 and 60 min, while this effect is gradually lost at 75 min of treatment. (**B**) Quantification of SGs. (**C**) KD of AR did not affect the level of different SG proteins. (**D**) Treatment with AR degrader, ARD-61, did not affect the level of SGs in response to ARPI stress. (**E**) Quantification of SGs. ARD-61 treatment reduced the level of AR protein as revealed by Western blot (**F**) without affecting the level of AR mRNA as revealed by qRT-PCR (**G**). The results are an average of three independent experiments with **P < 0.01. n.s., non-significant. Scale 10 μm.

Since AR mRNA phase-separated with YTHDF3 or G3BP1, we set out to define functional roles for AR mRNA in SG formation in response to ARPI stress. We found that AR silencing initially delayed SG formation in ARPI stressed cells, (Figure 7A and B), but with prolonged treatment, this effect was gradually lost. Additionally, the effect of AR KD on delaying SG formation was lost when the arsenite concentration was increased to >100 μM (Supplementary Figure S16), indicating that this effect is specific to ARPI stress (low concentration of arsenite and ENZA) condition. AR silencing did not affect levels of major SG proteins, including G3BP1, YTHDF3, YB-1 and CAPRIN1, excluding this as an effect on delayed SG formation (Figure 7C). These data suggest that while AR mRNA is not essential for SG formation, it may support SG formation via phase-separation with RBPs such as G3BP1 and YTHDF3. To further define roles of AR mRNA (not protein) in this process, LNCaP cells were treated with an AR degrader ARD-61 (50 nM for 6 h) (61). When ARD-61 treated cells were exposed to ARPI stress, SGs formed similar to untreated control cells (Figure 7D and E). Treatment with ARD-61 degraded AR protein levels by >99% (Figure 7F) without any significant change in AR mRNA levels (Figure 7G). Together, these observations - AR mRNA siRNA-reduced SG formation, and AR-protein degradation not affecting SG formation - suggest that AR mRNA (and not protein) support ARPI stress-induced SG formation. This observation prompted us to test whether other oncogenic transcription factors act similarly to AR mRNA. Silencing of estrogen receptor 1 (ESR1 or ERα) also delayed formation of SGs in MCF7 ER positive breast cancer cells after ER pathway inhibition (ERPI) stress (Supplementary Figure S17A). Similar to the AR protein, inhibition of ESR1 protein by either the degrader fulvestrant or LBD antagonist tamoxifen induced SG formation, suggesting that ESR1 mRNA and not its protein, support SG formation (Supplementary Figure S17B and C). ESR1 silencing did not affect levels of major SG proteins, including G3BP1, YTHDF3, YB-1 and CAPRIN1, excluding this as an effect on delayed SG formation (Supplementary Figure S17D). Together, these results suggest that mRNA transcripts of AR and ESR1 might support SG formation when their corresponding ligand-binding protein is antagonized or degraded.

### YTHDF3 and G3BP1 localize in spatially separate clusters within SGs

**Figure 8.**
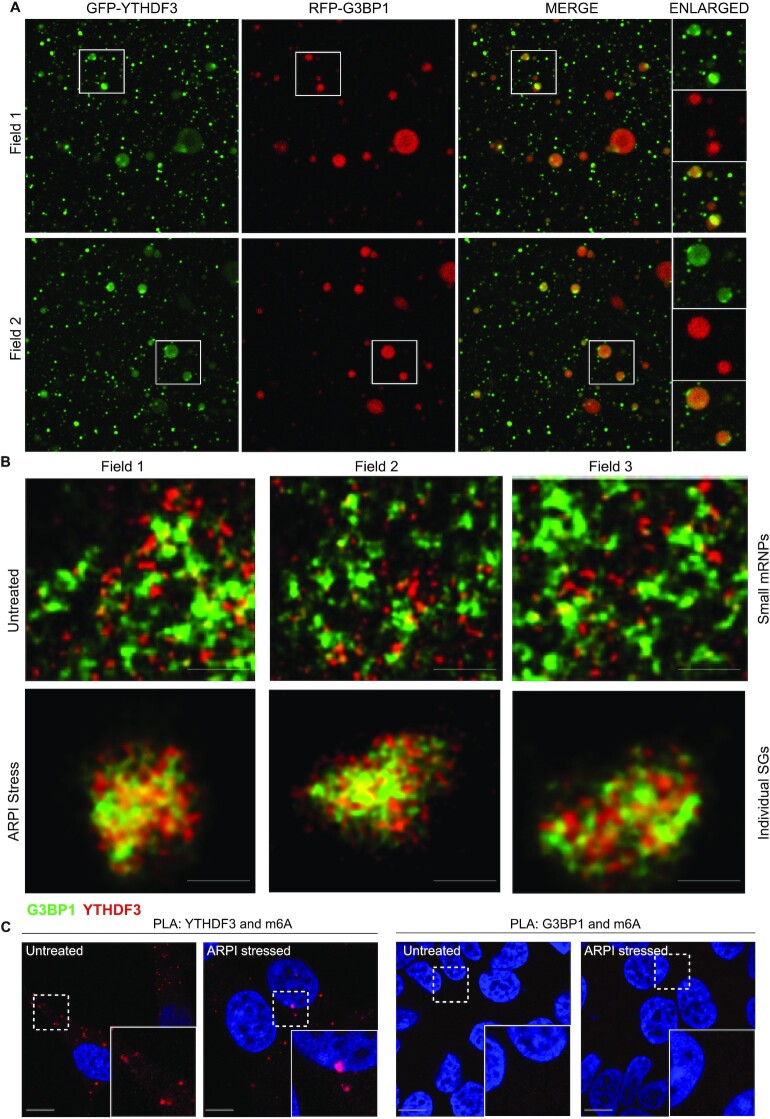
YTHDF3 and G3BP1 are present as separate clusters within the SGs. (**A**) GFP-YTHDF3 or RFP-G3BP1 recombinant proteins were mixed with total RNA from LNCaP to induce droplets. These droplets were then mixed together and viewed under 100x objective in a confocal microscope. Two imaging fields from independent experiments are shown. Part of the image is enlarged and shown on the right-side panels. Note that GFP-YTHDF3- and RFP-G3BP1 droplets did not mix completely but they were found as associated. (**B**) STED microscopy images showing the association of YTHDF3 and G3BP1 as separate clusters within the mRNPs in the unstressed cells, and within the SGs in ARPI stressed cells. Three images are shown. (**C**) PLA assay showing the association of YTHDF3 with m6A-modified mRNAs. G3BP1 showed reduced association with m6A-modified mRNAs.

SGs are complexes of several hundreds of RBPs comprised of a dense core linked with loosely packed regions termed as shells (62,63). SG core regions are mostly immobile and contain proteins like G3BP1 and TIA1 that are essential for SG formation. In contrast, the shell region is mostly dynamic and contain proteins like YTHDF family members that function to promote SG assembly (57,64). Since both YTHDF3 and G3BP1 are present in SGs, our results raise the possibility that SGs can recruit both m6A-modified and unmodified AR mRNA by associating with YTHDF3 or G3BP1, respectively. If so, two pools of m6A-modified and m6A-unmodified AR mRNA messages may co-exist as two separate YTHDF3 and G3BP1 clusters within SGs. We used in vitro droplet assay, in cell SG imaging using stimulated emission depletion (STED) microscopy, and proximity ligation assay (PLA) to define differential clustering of G3BP1 and YTHDF3, and their association with m6A-modified mRNAs. YTHDF3 was cloned in fusion with a green fluorescent protein (GFP), while G3BP1 was cloned in fusion with a red fluorescent protein (RFP). Both proteins were expressed in bacteria, purified as recombinant proteins (Supplementary Figure S7C and D), and subjected to phase-separation by mixing with total RNA. Phase-separated YTHDF3 and G3BP1 droplets were not completely miscible when mixed, forming separate clusters in the same complex (Figure 8A). These results support the notion that G3BP1 and YTHDF3 are present as individual clusters.

YTHDF3 and G3BP1 immunostaining using STED microscopy in unstressed and ARPI-stressed PCA cells also identified distinct clustering of YTHDF3 and G3BP1 proteins in unstressed cells that converge to form large SGs after ARPI stress. Similar to Figure 8A above, YTHDF3 and G3BP1 were immiscible and remained as distinct clusters within SGs (Figure 8B). Proximity ligation assay (PLA) (65) with antibodies against m6A and YTHDF3 or G3BP1 in unstressed or ARPI-stressed cells was positive for YTHDF3-m6A association while G3BP1-m6A association was negative (Figure 8C). Collectively, our results suggest that m6A-unmodified AR messages phase separate with G3BP1 in SG cores, while m6A-modified AR messages phase separate with YTHDF3 in the more dynamic SG shell.

### M6A-modification of AR mRNA regulates its translation

**Figure 9.**
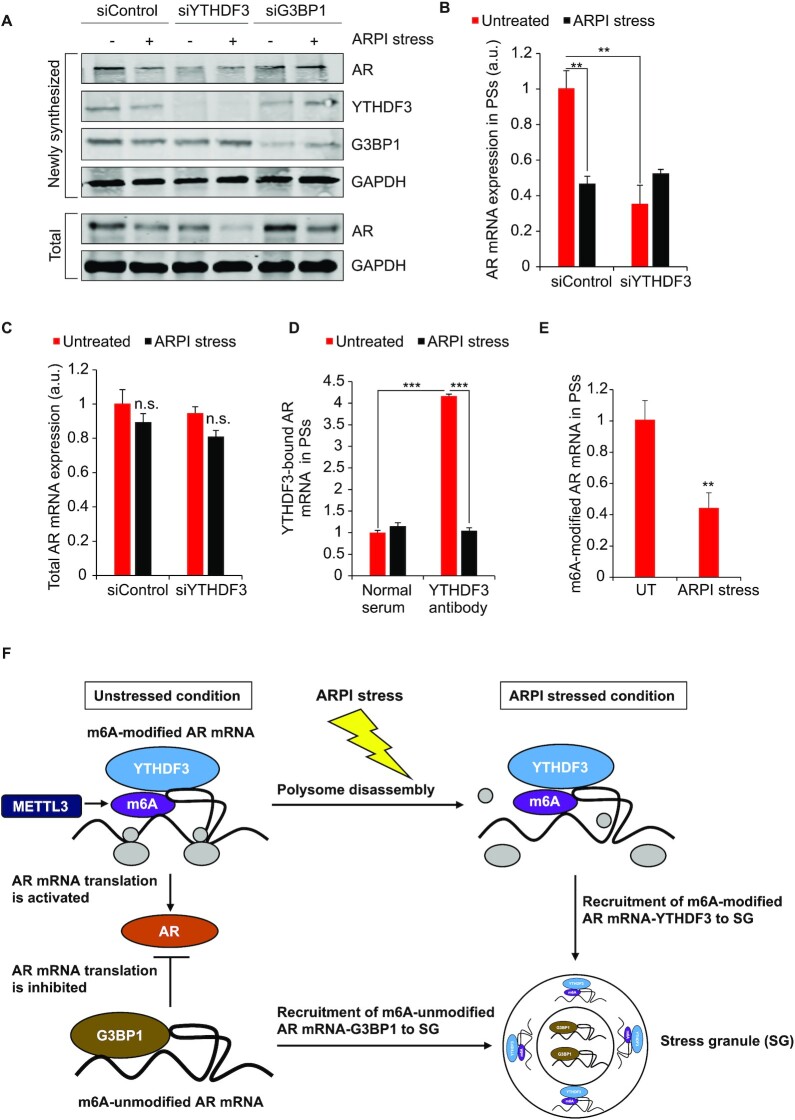
YTHDF3 translationally regulates AR mRNA. (**A**) Knockdown (KD) of G3BP1 did not affect rate of synthesis of AR protein, while KD of YTHDF3 reduced the rate of synthesis of AR protein as measured by Click chemistry (see Methods for details). (**B**) KD of YTHDF3 reduced the level of AR mRNA in the PSs. (**C**) KD of YTHDF3 did not affect the total mRNA level of AR. (**D**) YTHDF3 binds to AR mRNA in the PSs. (**E**) m6A-modified AR mRNA is reduced in the PSs after ARPI stress. Results are presented as an average of 3 independent experiments with ***P < 0.001, **P < 0.01, n.s., non-significant. (**F**) Model illustrating the regulation of AR mRNA during ARPI stress. Under the unstressed condition, AR mRNA is present as two fractions. m6A-modified translatable AR mRNA fraction, regulated by METTL3, is associated with YTHDF3 in the PSs, and m6A-unmodified untranslatable AR mRNA fraction is associated with G3BP1 in the non-PS fraction. When the cells are exposed to ARPI stress, PSs disassemble, and the m6A-modified AR mRNA and associated YTHDF3 is redistributed from the PSs to SGs to form the AR mRNA-YTHDF3 cluster. At the same time, G3BP1 associated m6A-unmodified AR mRNA also redistributes from the non-PS fraction to SGs to form the AR mRNA-G3BP1 cluster. Thus, these two ribonucleoprotein (RNP) clusters, AR mRNA-YTHDF3 and AR mRNA-G3BP1, co-exist in the SG.

G3BP1 binding with m6A-unmodified fraction of AR mRNA might constitute an untranslatable AR mRNA pool, a postulate consistent with prior reports that G3BP1 associates with an untranslatable pool of cellular mRNAs (66), and data in Supplementary Figure S18 (upper panel) showing that G3BP1 is barely detectable in PS fractions and accumulates mainly in the untranslated non-PS fractions. Since G3BP1 was not associated with PSs and is enriched in untranslated fractions, G3BP1 KD did not affect the rate of AR protein synthesis as measured by click chemistry (Figure 9A) (42). On the other hand, YTHDF3 is enriched in both PS and non-PS fractions in untreated cells, and following ARPI stress YTHDF3 is redistributed from PSs to untranslated non-PS fractions (Supplementary Figure S18, middle panel). Therefore, contrary to G3BP1 KD, YTHDF3 silencing reduced protein levels of AR (Figure 9A) and mRNA in PSs (Figure 9B), without affecting total AR mRNA (Figure 9C) or AR protein degradation rates (Supplementary Figure S19). Pulldown of YTHDF3-associated mRNAs using riboimmunoprecipitation (RIP) confirmed AR mRNA interaction with YTHDF3 in the PSs (Figure 9D). Additionally, pulldown of m6A-modified mRNAs using anti-m6A antibodies demonstrated enrichment of m6A-modified AR mRNA in PSs in unstressed cells (Figure 9E). After ARPI stress, m6A-modified AR mRNA level is reduced in the PSs and is redistributed from the PSs to non-PS fractions (Figure 9E; Supplementary Figure S20). Together, this data suggests that in unstressed cells YTHDF3 binds m6A-modified AR mRNA and stimulates its translation in PSs (model in Figure 9F), consistent with previous reports on YTHDF3-mediated translation of m6A-modified mRNAs in the PSs (67,68). Our model (Figure 9F) also indicates that the total amount of m6A AR mRNA-YTHDF3 complexes is not affected by ARPI stress (Figure 4C), rather ARPI stress triggers redistribution of m6A AR mRNA-YTHDF3 from actively translating PSs to untranslated fraction including SGs, leading to reduced protein translation.

Since many residues are m6A-modified in AR mRNA (Figure 4A), we explored whether AR mRNA translation can be affected by altering its m6A modifications. KD of m6A methylase METTL3 using siRNAs reduced AR protein levels (Supplementary Figure S21A). In contrast, KD of other m6A modifying enzymes including WTAP, KIAA1429 (VIRMA), and METTL14 did not affect the protein level of AR, suggesting that METTL3 specifically regulates the protein expression of AR (Supplementary Figure S21A). Total AR mRNA levels were not significantly changed in siMETTL3 KD cells compared to control cells, indicating that MTETTL3 does not transcriptionally regulate AR mRNA (Supplementary Figure S21B). Furthermore, while total m6A-modified AR mRNA was slightly reduced (Supplementary Figure S21C), PS-associated m6A-modified AR mRNA levels were reduced by >50% (Supplementary Figure S21D) in METTL3 KD cells compared to control cells. These observations indicate that METTL3 can methylate AR mRNA associated with PSs, and the reduced AR protein expression in METTL3 KD cells results from lower PS levels of m6A-modified AR mRNAs. To analyse the effect of METTL3 on YTHDF3-regulated AR mRNA translation, we have compared AR mRNA-YTHDF3 interactions in PSs in control cells versus METTL3 KD cells. We found that KD of METTL3 significantly reduced the association of AR mRNA with YTHDF3 in PSs in both ARPI stress and untreated conditions (Supplementary Figure S21E). This indicates that when methylation of AR mRNA is reduced, AR mRNA association with YTHDF3 in PSs is also reduced, which in turn reduces AR mRNA translation (model in Figure 9F). Even though METTL3 KD affected the translation of AR mRNA, it did not affect the formation of SGs in response to ARPI stress (Supplementary Figure S21F), suggesting that the changes in the m6A modification of AR might not have a significant effect on SG formation.

Since METTL3 and YTHDF3 regulate AR mRNA translation, we checked whether downregulating the expression of these proteins could reduce AR downstream activity in LNCaP cells. AR transactivation assay (45) showed a significant reduction in AR activity in YTHDF3 KD cells compared to control cells (Supplementary Figure S22A). Interestingly, G3BP1 KD also reduced AR activity, albeit to a lesser extent compared to YTHDF3 KD cells, indicating that G3BP1 might affect AR downstream activities independent of AR protein levels, perhaps via G3BP1’s broader role in stress response (Supplementary Figure S22A). We also found significantly reduced AR activity in METTL3 KD cells compared to control cells (Supplementary Figure S22B). Together, these results suggest that METTL3-regulated m6A modification of AR mRNA and m6A-modified AR mRNA-YTHDF3 interaction in PSs supports AR mRNA translation and increases AR downstream signaling.

### G3BP1 and YTHDF3 are cytoprotective in ARPI stressed cells

**Figure 10.**
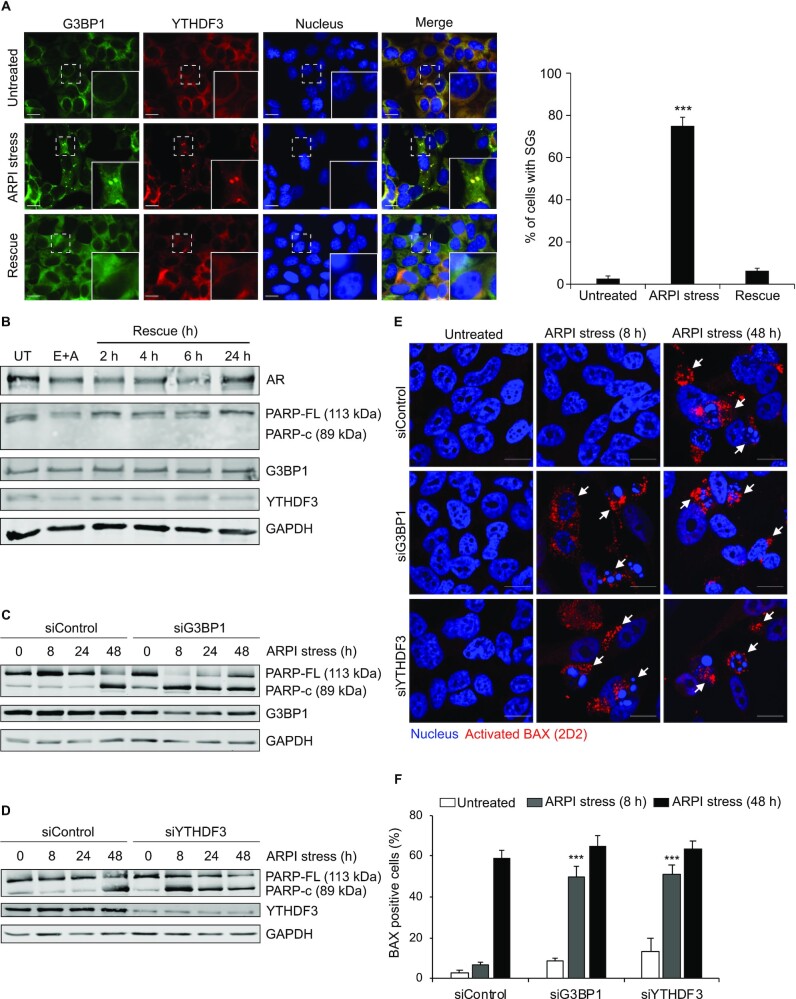
G3BP1 and YTHDF3 are cytoprotective proteins. (**A**) ARPI stress induced SG formation is reversible. LNCaP cells were unstressed, ARPI stressed or rescued from treatment. The cells were then stained with anti-G3BP1 and anti-YTHDF3 antibodies to reveal the SGs. Quantification of SGs is shown on the right-side panel. Note that ARPI stress- induced SG formation was reversed when treatment was withdrawn. (**B**) ARPI stress induced reduction in AR protein was restored to steady state level following stress removal. (C–F) KD of G3BP1 or YTHDF3 reduced cell survival in LNCaP cells. LNCaP cells transfected with siControl, siG3BP1 or siYTHDF3 were unstressed or treated with ARPI stress for 8, 24 and 48 h. The cell lysates were subjected to Western blotting for the indicated antibodies. Note that ARPI stress enhanced PARP cleavage in siG3BP1 (**C**) and siYTHDF3 (**D**) cells compared to control cells (8 h treatment). The above cells were subjected to IF using antibodies against activated BAX (2D2), an indicator of apoptosis (**E**). BAX positive cells are indicated by arrowheads. (**F**) Quantification of BAX activation. Note that acute ARPI stress strongly activated BAX in siG3BP1 and siYTHDF3 cells compared to control cells (8 h treatment). The results are an average of three independent experiments with ***P < 0.001. Scale 10 μm.

**Figure 11.**
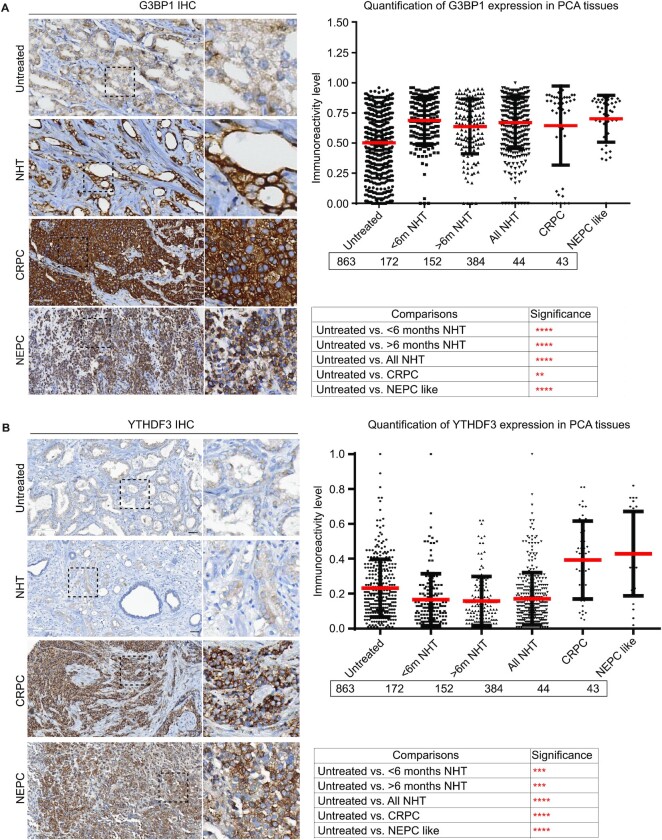
Immunohistochemistry (IHC) analysis of G3BP1 and YTHDF3 in PCA TMAs. PCA tissue microarrays (TMAs) were stained with anti-G3BP1 (**A**) and anti-YTHDF3 (**B**) antibodies. A part of the image is enlarged and shown. Quantification of staining is shown on the right-side panels. Statistics of quantification data is presented below respective graphs. Note that G3BP1 expression is increased in ARPI stressed, CRPC and NEPC tissues, while YTHDF3 expression is enhanced in CRPC and NEPC tissues. NHT: neoadjuvant hormonal therapy; CRPC: castration resistant PCA; NEPC: neuroendocrine PCA.

SGs form acutely after ARPI stress, and disassemble when stress is removed (Figure 10A). Similarly, decreases in AR protein expression after ARPI stress return to baseline within 24 h after cells recover from stress (Figure 10B). We did not detect significant apoptosis under the conditions of acute ARPI stress when SGs are induced and AR protein levels decrease (Figure 10B). ARPI stress also downregulated global protein synthesis in LNCaP cells as measured by AHA-labelling-Click experiments (Supplementary Figure S23A). We previously reported that this could be linked to recruitment of mRNAs encoding pro-apoptotic proteins to SGs, with selective translation of survival factors (16). These results suggest that acute ARPI stress-induced SG formation, triggered by AR mRNA, are dynamic stress-dependent processes that may activate cytoprotective pathways. We and others have defined G3BP1 (16,31,69,70) and YTHDF3 (71) as survival factors in cancer. Consistent with this premise, G3BP1 or YTHDF3 silencing sensitized LNCaP cells to acute ARPI stress (8 h) as evidenced by enhanced PARP cleavage (Figure 10C and D) and BAX activation (by 2D2 antibody detection of oligomerized BAX) (Figure 10E and F), compared to ARPI-stressed control cells. With prolonged chronic ARPI stress (48 h), control cells were also affected and showed enhanced PARP processing and BAX activation. When stress treatment was withdrawn, the siControl cells resumed growth similar to those cells that were never stressed, consistent with our findings that the ARPI stress-induced changes are dynamic and reversible (Supplementary Figure S23B and C). Notably, the growth profile of siG3BP1 or siYTHDF3 cells did not reverse, indicating that both RBPs are cytoprotective during stress, and thereby may support treatment resistance to ARPI stress (Supplementary Figure S23B and C). Indeed, G3BP1 is expressed at higher levels in patient samples of post-ARPI-treated, CRPC, and neuroendocrine prostate cancer (NEPC) (Figure 11A). YTHDF3 protein expression decreases slightly after short term ARPI, but increases dramatically in CRPC and NEPC tissues (Figure 11B). These results link ARPI-induced increases in G3BP1 and YTHDF3 to cytoprotection and treatment resistance, and support further studies targeting these RBPs to sensitize PCA cells to ARPI.

## DISCUSSION

The AR is the key driver and most important therapeutic target for advanced PCA (72,73). Treatment resistance via adaptation and clonal selection occurs through transcriptional, epigenetic, and mutational changes (74,75), and while recent landscape papers are defining these genomic structural and epigenetic alterations in CRPC (12,13,76), enormous barriers still exist to integrate this information with functional determinants of tumour biology. Emerging evidence, including our work, suggests that stress adaptation occurs through changes in mRNA translation. For instance, under acute stress, tumor cells suppress overall mRNA translation, sequester translationally repressed mRNAs encoding pro-apoptotic proteins in SGs, and selectively translate mRNAs encoding survival factors for cellular protection (16,31,77). In this paper, we define novel interactions between AR mRNA and two RBPs that trigger SG formation, regulate AR mRNA translation, and promote PCA cell survival. We focus on acute rather than chronic responses, as rapid adaptation is critical for survival under treatment stress, and the subsequent emergence of resistance.

We observed induction of SGs in ARPI-stressed AR^+^ PCA cell lines co-incident with reduced AR protein levels. Immuno-staining levels of SGs increased, while AR protein levels decreased, in post-ARPI treated PCA tissues, credentialing this stress response in clinical specimens. Total AR mRNA or protein degradation rates were not altered in ARPI stressed cells; rather the reduced AR protein expression was due to reduced AR mRNA translation as AR messages in the PSs were disassembled and recruited to SGs in ARPI-stressed cells. We defined m6A modification of AR mRNA as a key determinant for its partitioning between PSs and SGs, guided by its differential interaction with YTHDF3 and G3BP1. G3BP1 tends to not bind m6A-modified transcripts (53,78), while YTHDF3 is a strong binder for m6A-modified transcripts (67). Additionally, both G3BP1 and YTHDF3 undergo liquid-liquid phase-separation (LLPS) with RNA to form liquid droplets (44,58). We identified both m6A-modified and m6A-unmodified AR mRNA fractions inside cells. YTHDF3 binds m6A-modified AR mRNAs that are actively translating in the PSs, and YTHDF3 silencing reduced m6A-modified AR mRNA levels in PSs, leading to reduced AR protein synthesis. These data are consistent with published reports of YTHDF3 binding to m6A-containing mRNAs to promote their translation efficiency (67,68,79–81). In contrast, G3BP1 bound an untranslatable fraction of m6A-unmodified AR mRNA, and G3BP1 KD did not affect AR protein levels.

Following ARPI stress, levels of m6A-modified AR mRNA decrease, reflecting either lower m6A-writer or enhanced m6A-eraser activity (82). Under ARPI stress, m6A-modified AR mRNAs disassemble from PSs, phase-separate as YTHDF3 droplets, and cluster in SGs, supporting the notion that reduction in expression of AR reflects disassembly of m6A-modified AR mRNAs in the PSs of ARPI stressed cells. The m6A-unmodified AR mRNA fraction phase-separated as G3BP1 droplets formed a cluster in SGs distinct from that of YTHDF3. Further characterization defined two pools of AR mRNAs based on m6A modification, one interacting with YTHDF3, and the other with G3BP1, and these two distinct fractions form separate immiscible clusters within SGs based on their m6A-modification. The above studies suggest that the percentage of m6A-modification is a key determinant in AR mRNA translation and its clustering with G3BP1 or YTHDF3. METTL3 silencing reduced m6A levels of AR mRNA in PSs, AR mRNA-YTHDF3 interaction, AR protein levels, and AR transactivation, illustrating how m6A regulatory writers can affect AR downstream signaling. METTL3 KD reduces AR mRNA translation without affecting SG formation, suggesting m6A modification of AR mRNA might not be a significant regulator of SG formation. Other methylases that add m6A’s to AR mRNA may regulate its shuttling between PSs and SGs, or alternatively, demethylases (e.g. ALKBH5) could be activated by ARPI stress and remove m6A from AR mRNA to accelerate the phase separation of m6A-unmodified AR mRNA with G3BP1 and SG formation. Future studies are required to characterise other methylases that control AR mRNA m6A modifications and AR protein translation.

SGs adopt heterogeneous structures formed by initial nucleation of the G3BP1-rich cores in juxtaposition with a more dynamic shell (83,84). YTHDF3 clusters tend to reside in the periphery and at the junctions connecting G3BP1 clusters (64), which may function to promote SG formation by coalescing small G3BP1 core clusters into larger granules. SGs are formed by the LLPS of hundreds of proteins, which exist as multiple clusters within SGs. These different clusters contain distinct combinations of mRNPs, i.e. specific set of RBPs with their associated mRNAs (85). Different clusters can be spatially organized within the SGs to achieve both structural integrity as well as dynamic shuttling of SG components (64,86). Our data suggests that m6A-modified AR mRNA associated with YTHDF3 clusters in the SG shell dynamically shuttle with PSs during stress. In contrast, m6A-unmodified AR mRNA within G3BP1 clusters provide structural integrity to SGs and are translationally incompetent (see model in Figure 9F).

RNA and its modifications, including m6A, are critical elements for the size and composition of phase-separated RNA–protein condensates (25,57,64). Messages encoding proteins such as ACTB, HSPA8, PABPC1 and EEF2 are strong triggers for in vitro LLPS of G3BP1 (43), indicating that mRNAs of different genes can modulate the phase-separation of SG-associated proteins. Interestingly, we found that silencing of AR mRNA in LNCaP cells delayed the formation of SGs, suggesting AR mRNA supports SG assembly in ARPI-stressed cells. Interestingly, depleting mRNA encoding another oncogenic transcription factor, ESR1, also delayed SG formation, suggesting that oncogenic transcription factors AR and ESR1 might function to phase-separate their respective mRNAs when their corresponding hormone activation pathway is blocked.

Since SGs are dynamic complexes of hundreds of proteins and mRNAs, it is reasonable that other RBPs, in addition to YTHDF3 and G3BP1, are involved in regulating the translation of AR mRNA, and its association with SGs. In fact, a recent study reports that DDX3 regulates AR mRNA translation by binding AR mRNA and sequestering it to SGs, which reduces AR mRNA translation (87). These findings indicate that AR mRNA can interact with RBPs like DDX3 in addition to G3BP1 and YTHDF3. Other groups have also reported that m6A demethylase ALKBH5 is directly regulated by DDX3, leading to decreased m6A methylation of specific mRNAs (88,89). Therefore, RBPs such as G3BP1, YTHDF3, and DDX3 might work in tandem with m6A demethylase ALKBH5 to influence AR mRNA translational repression and SG association in ARPI stressed cells.

ARPI stress-induced SG formation and suppression of AR expression is dynamic and reversible, with SG disassembly coinciding with AR expression returning to baseline levels after treatment withdrawal. These oscillations of AR protein expression might be linked to the dynamic shuttling of AR mRNA between PSs and SGs. While small amounts of AR protein are translated after ARPI stress, possibly through selective mRNA translation of a part of m6A-modified AR mRNA in PSs or in SGs (90), this is insufficient to protect G3BP1 or YTHDF3 silenced LNCaP cells to ARPI stress. We also found a higher expression of YTHDF3 and G3BP1 in highly resistant CRPC and specific NEPC tissues, consistent with prior studies reporting higher levels of G3BP1 and YTHDF3 in different tumor tissues, and are negatively prognostic (31,70,91–94).

Using context-dependent and physiologically relevant anti-cancer stress in PCA or breast cancer cells, this study shows that AR or ER mRNA support the formation of SGs under specific stress of AR or ER antagonism. Other approved therapeutics, docetaxel or olaparib, did not induce SGs. While induction of SGs occurs at higher non-physiological concentrations of oxidative, proteotoxic, or metabolic stressors as a general stress response (31), our data highlight the importance of modelling physiologically relevant and context-specific stress conditions for investigating SGs in selected cancers. We also observed global translational inhibition, and activation of survival pathways (data not shown) in response to ARPI stress, consistent with our prior reports of PS-mediated enhanced translation of mRNAs encoding cell survival factors, with SG-sequestration of mRNAs encoding pro-apoptotic proteins (16). A cumulative effect of these molecular processes - reduced AR expression, global repression in translation, and activation of survival pathways - support PCA cell stress adaptation, survival, and treatment resistance.

In conclusion, cytoprotective SGs are formed after acute ARPI stress in PCA cell lines and tissues. The RBPs YTHDF3 and G3BP1 coordinately regulated translation of AR mRNA, with YTHDF3 binding m6A-modified, translationally active AR mRNA in PSs, and G3BP1 binding m6A-unmodified, translationally repressed AR mRNA. Under acute ARPI stress, AR mRNA supported the phase separation and SG formation - m6A-unmodified AR mRNA bound to G3BP1 phase-separated to a core cluster in SGs, while YTHDF3- bound m6A-modified AR mRNA localized as a distinct shell cluster surrounding the SG core. ARPI-induced SG formation reduced global translation, while activating adaptive cytoprotective pathways; blocking this adaptive response by G3BP1 or YTHDF3 silencing sensitized PCA cells to ARPI stress. Our research defines a novel mechanism of AR mRNA translation involving AR mRNA m6A regulatory proteins and SG formation that may be exploitable in designing ARPI-based therapies.

## DATA AVAILABILITY

The data that support the findings of this study are available from the corresponding author, upon reasonable request.

## Supplementary Material

gkab1247_Supplemental_Files
