# Supplementary material for: Regulation of AR mRNA translation in response to acute AR pathway inhibition
Source: Nucleic Acids Res. 2021 Dec 23;50(2):1069–91. doi: 10.1093/nar/gkab1247 (PMC8789049; doi:10.1093/nar/gkab1247)
Supplement: gkab1247_Supplemental_Files [file gkab1247_supplemental_files.zip › Other supplementary materials legends NAR-03196-V-2021.docx]

**Other supplementary materials legends**

**Supplementary Table 1.** Sequences of oligos used for EMSA, phase separation, and qRT-PCR.

**Movie 1.** Movie showing the dynamics of phase separated G3BP1 droplets.

**Movie 2.** Movie showing the dynamics of phase separated YTHDF3 droplets.
