# Supplementary material for: Regulation of AR mRNA translation in response to acute AR pathway inhibition
Source: Nucleic Acids Res. 2021 Dec 23;50(2):1069–91. doi: 10.1093/nar/gkab1247 (PMC8789049; doi:10.1093/nar/gkab1247)
Supplement: gkab1247_Supplemental_Files [file gkab1247_supplemental_files.zip › Somasekharan et al Supplementary Figures NAR-03196-V-2021.pdf]

Fig. S1

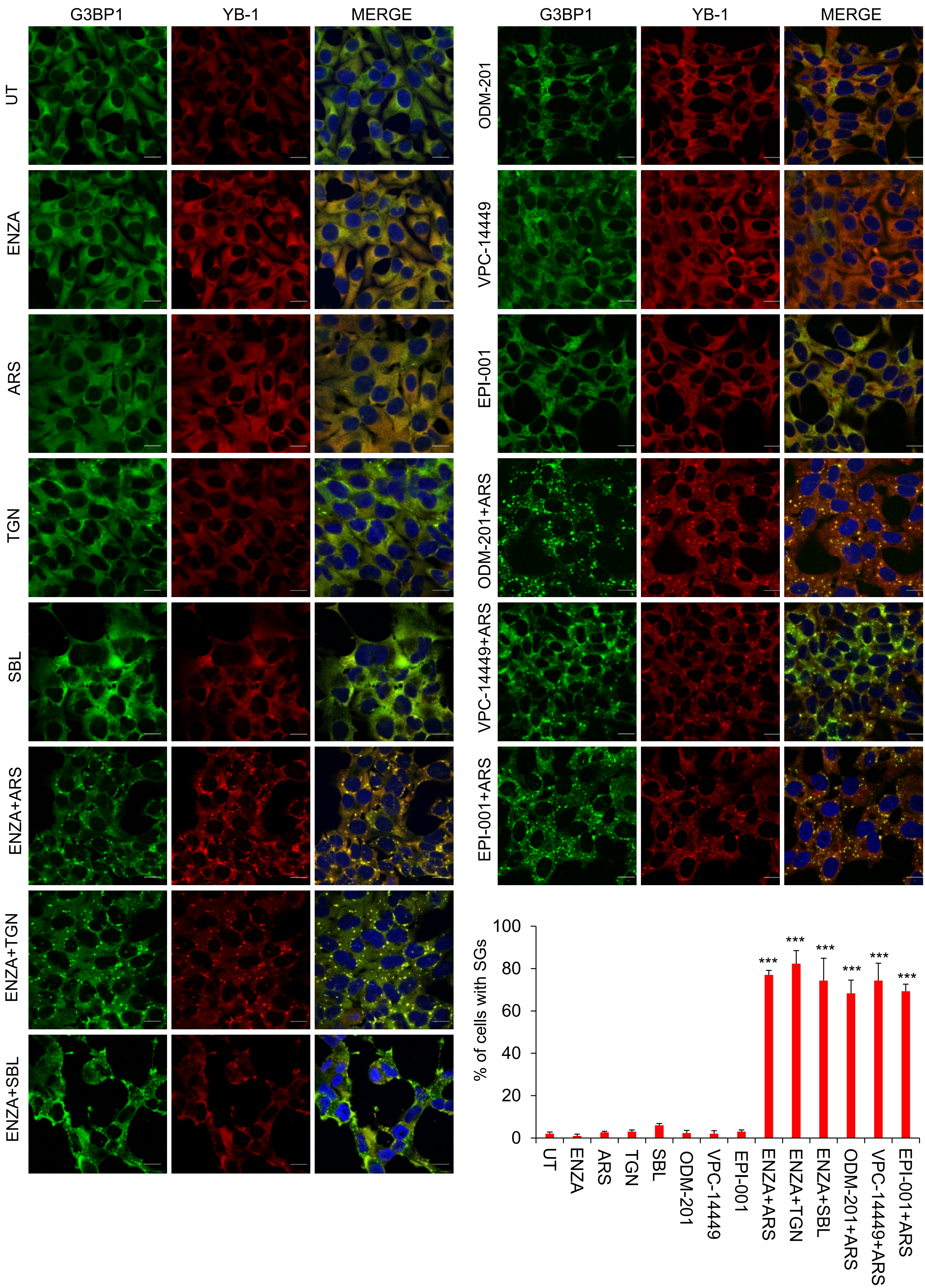

G3BP1
YB-1
MERGE
UT
ENZA
ARS
TGN
SBL
ENZA+ARS
ENZA+TGN
ENZA+SBL
ODM-201
VPC-14449
EPI-001
ODM-201+ARS
VPC-14449+ARS
EPI-001+ARS
% of cells with SGs
0
20
40
60
80
100
***

Fig. S2

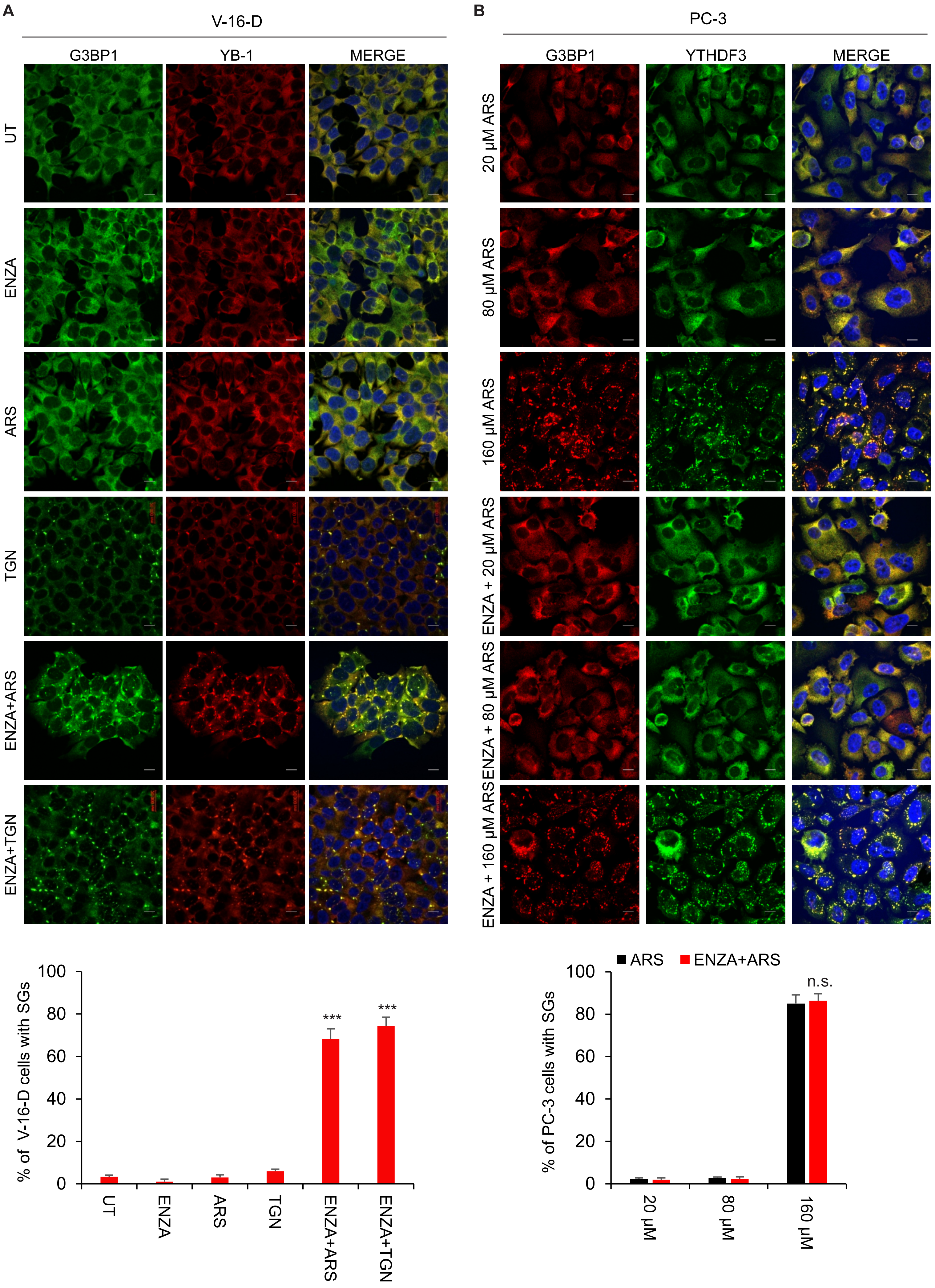
A
V-16-D
G3BP1
YB-1
MERGE
UT
ENZA
ARS
TGN
ENZA+ARS
ENZA+TGN
% of V-16-D cells with SGs
100
80
60
40
20
0
***
***
UT
ENZA
ARS
TGN
ENZA+ARS
ENZA+TGN
B
PC-3
G3BP1
YTHDF3
MERGE
20 μM ARS
80 μM ARS
160 μM ARS
ENZA + 20 μM ARS
ENZA + 80 μM ARS
ENZA + 160 μM ARS
ARS
ENZA+ARS
n.s.
% of PC-3 cells with SGs
100
80
60
40
20
0
20 μM
80 μM
160 μM

Fig. S3

A

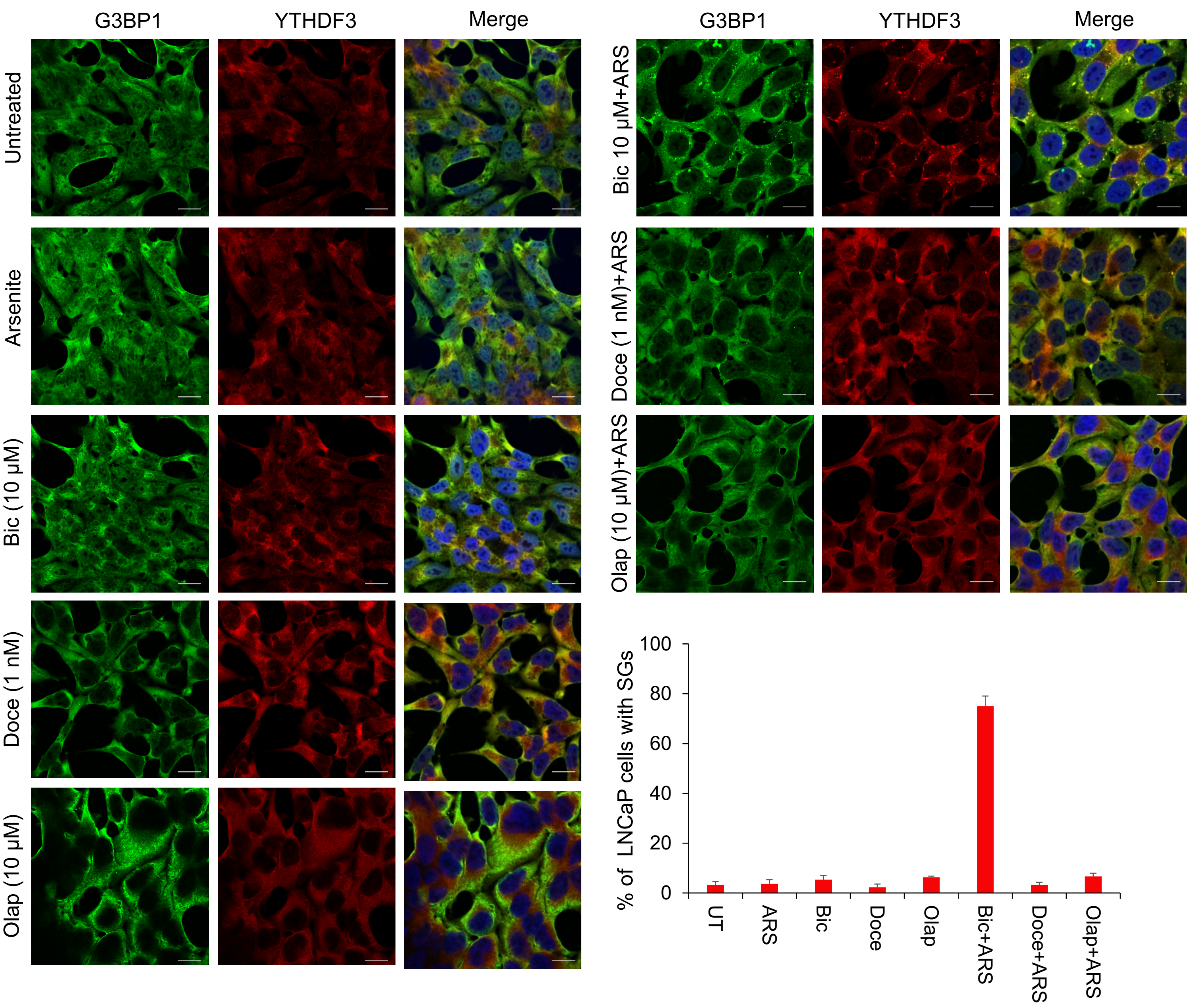
G3BP1
YTHDF3
Merge
Untreated
Arsenite
Bic (10 μM)
Doce (1 nM)
Olap (10 μM)
Bic 10 μM+ARS
Doce (1 nM)+ARS
Olap (10 μM)+ARS
% of LNCaP cells with SGs
100
80
60
40
20
0
UT
ARS
Bic
Doce
Olap
Bic+ARS
Doce+ARS
Olap+ARS

B

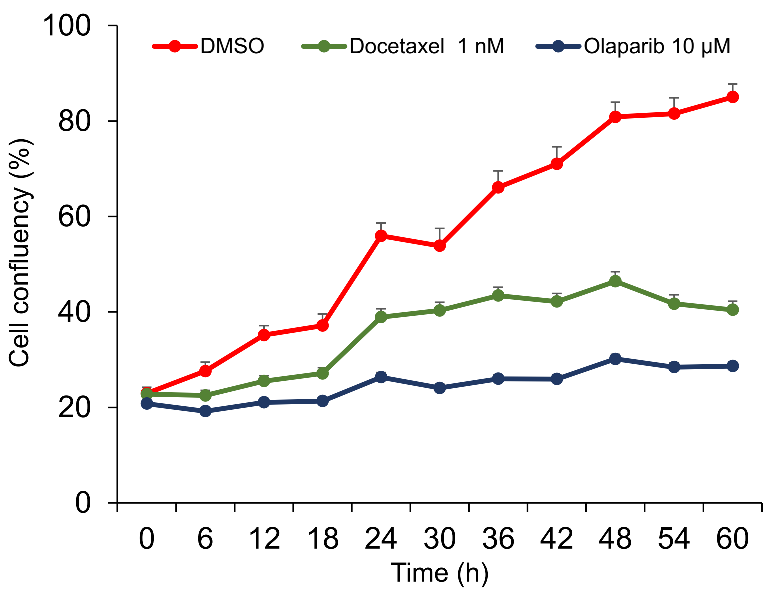
DMSO
Docetaxel 1 nM
Olaparib 10 μM
Cell confluency (%)
100
80
60
40
20
0
0
6
12
18
24
30
36
42
48
54
60
Time (h)

Fig. S4

A

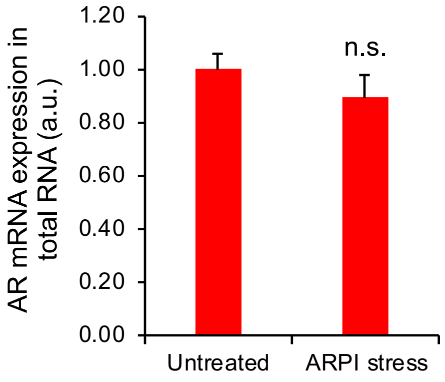

AR mRNA expression in total RNA (a.u.)
1.20
1.00
0.80
0.60
0.40
0.20
0.00
n.s.
Untreated
ARPI stress

B

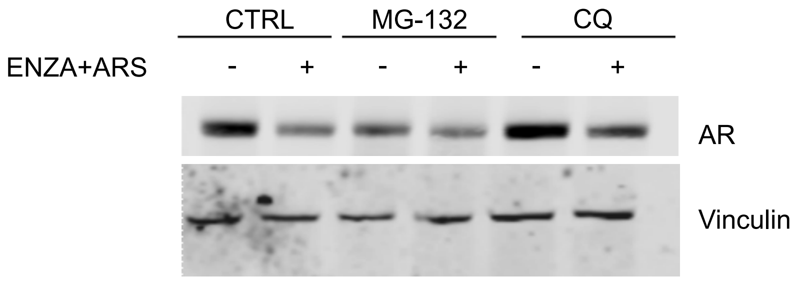

CTRL
MG-132
CQ
ENZA+ARS
- + - + - +
AR
Vinculin

Fig. S5

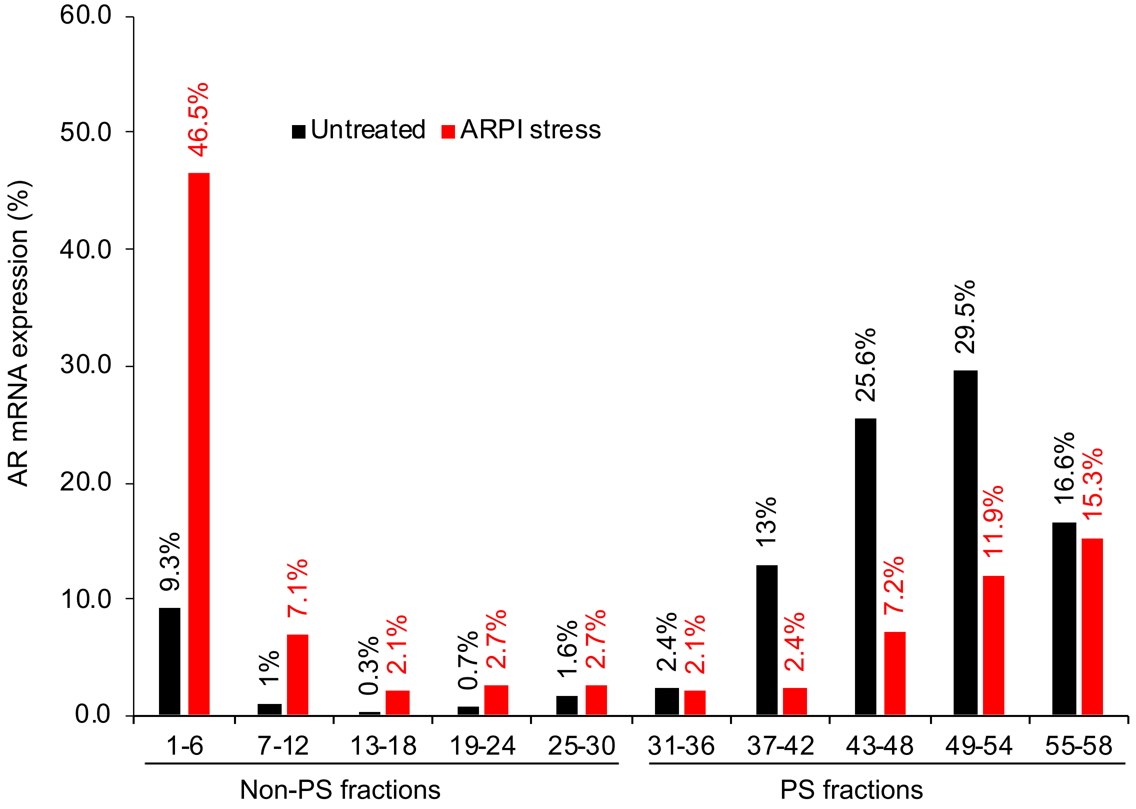

AR mRNA expression (%)
60.0
50.0
40.0
30.0
20.0
10.0
0.0
Untreated
ARPI stress
9.3%
46.5%
1%
7.1%
0.3%
2.1%
0.7%
2.7%
1.6%
2.7%
2.4%
2.1%
13%
2.4%
25.6%
7.2%
29.5%
11.9%
16.6%
15.3%
1-6
7-12
13-18
19-24
25-30
31-36
37-42
43-48
49-54
55-58
Non-PS fractions
PS fractions

Fig. S6

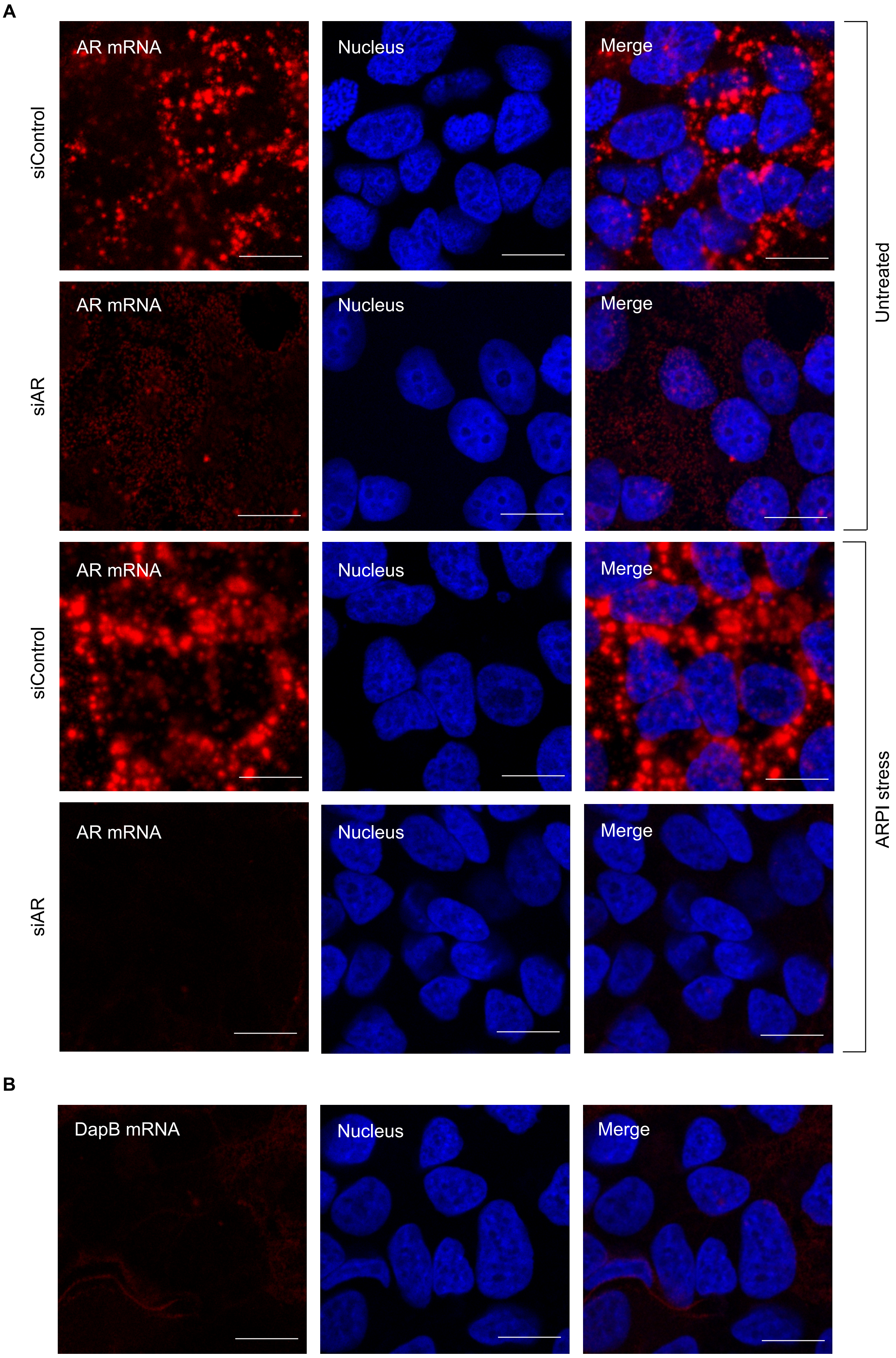
A
AR mRNA
Nucleus
Merge
siControl
siAR
siControl
siAR
Untreated
ARPI stress
B
DapB mRNA
Nucleus
Merge

Fig. S7

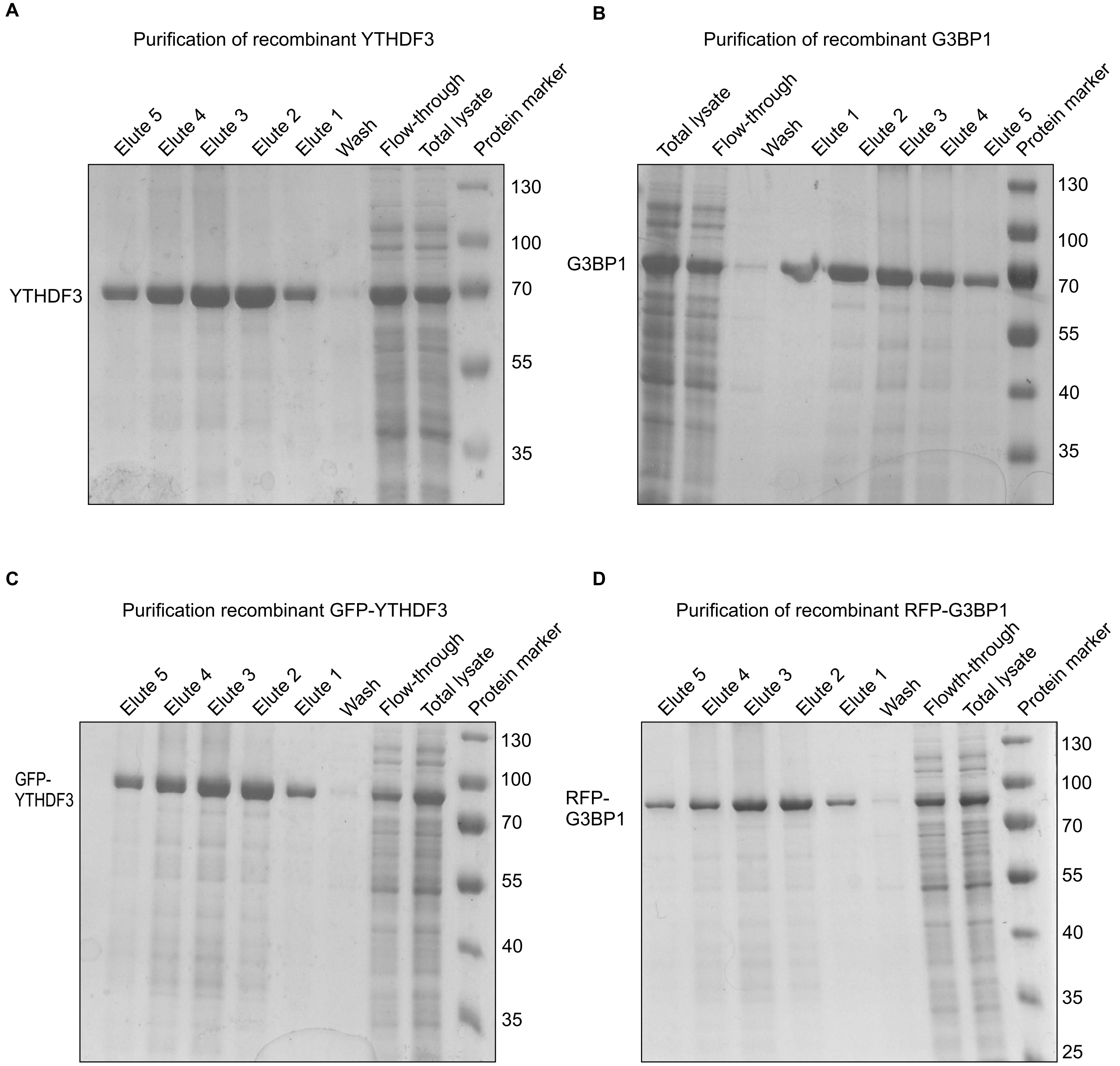
A
Purification of recombinant YTHDF3
Elute 5
Elute 4
Elute 3
Elute 2
Elute 1
Wash
Flow-through
Total lysate
Protein marker
YTHDF3
130
100
70
55
35
B
Purification of recombinant G3BP1
Total lysate
Flow-through
Wash
Elute 1
Elute 2
Elute 3
Elute 4
Elute 5
Protein marker
G3BP1
130
100
70
55
40
35
C
Purification recombinant GFP-YTHDF3
Elute 5
Elute 4
Elute 3
Elute 2
Elute 1
Wash
Flow-through
Total lysate
Protein marker
GFP-
YTHDF3
130
100
70
55
40
35
D
Purification of recombinant RFP-G3BP1
Elute 5
Elute 4
Elute 3
Elute 2
Elute 1
Wash
Flowth-through
Total lysate
Protein marker
RFP-
G3BP1
130
100
70
55
40
35
25

Fig. S8

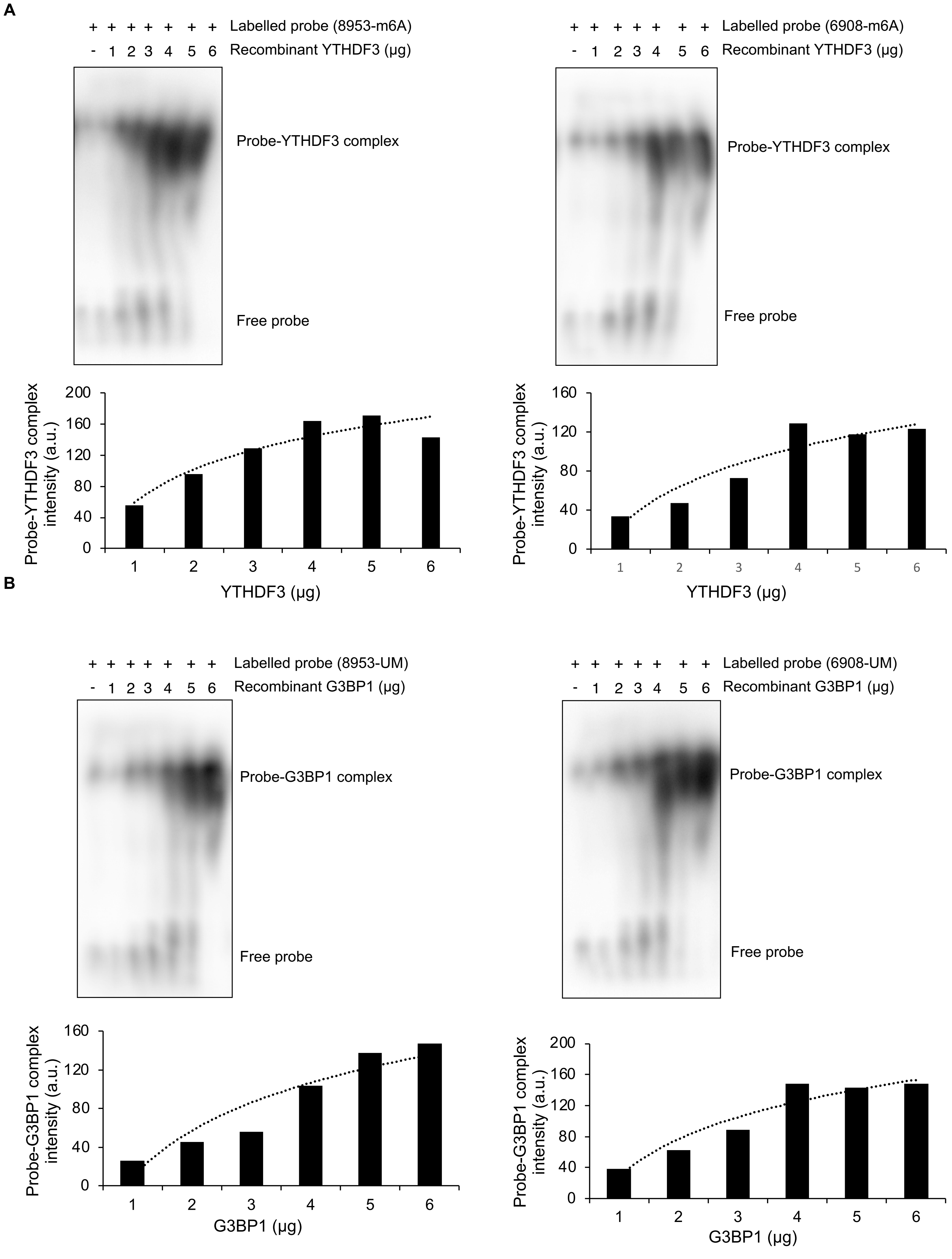

A
+ + + + + + + Labelled probe (8953-m6A)
- 1 2 3 4 5 6 Recombinant YTHDF3 (µg)
Probe-YTHDF3 complex
Free probe
Probe-YTHDF3 complex intensity (a.u.)
200
160
120
80
40
0
1 2 3 4 5 6
YTHDF3 (µg)
+ + + + + + + Labelled probe (6908-m6A)
- 1 2 3 4 5 6 Recombinant YTHDF3 (µg)
Probe-YTHDF3 complex
Free probe
Probe-YTHDF3 complex intensity (a.u.)
160
120
80
40
0
1 2 3 4 5 6
YTHDF3 (µg)
B
+ + + + + + + Labelled probe (8953-UM)
- 1 2 3 4 5 6 Recombinant G3BP1 (µg)
Probe-G3BP1 complex
Free probe
Probe-G3BP1 complex intensity (a.u.)
160
120
80
40
0
1 2 3 4 5 6
G3BP1 (µg)
+ + + + + + + Labelled probe (6908-UM)
- 1 2 3 4 5 6 Recombinant G3BP1 (µg)
Probe-G3BP1 complex
Free probe
Probe-G3BP1 complex intensity (a.u.)
200
160
120
80
40
0
1 2 3 4 5 6
G3BP1 (µg)

Fig. S9

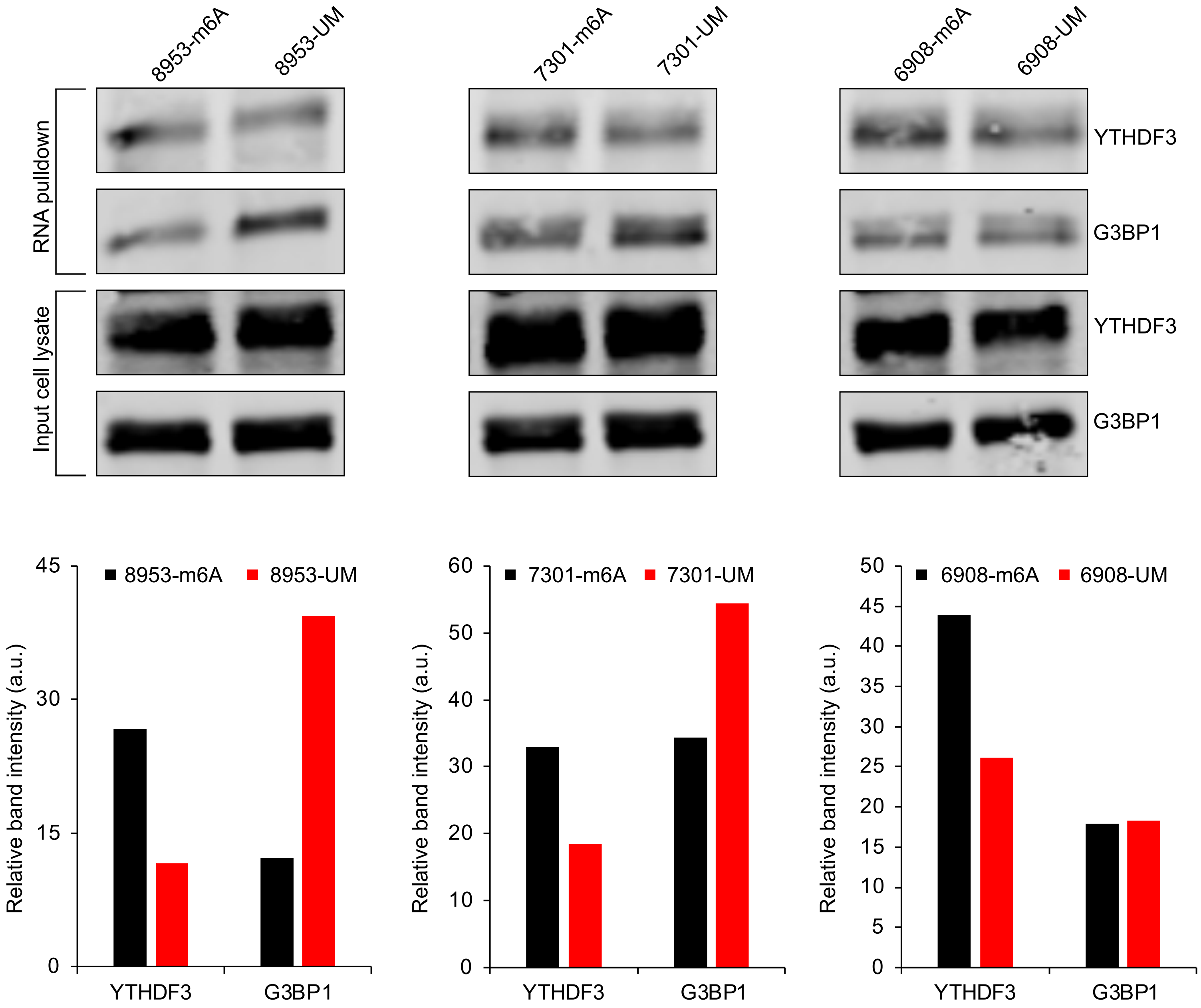
8953-m6A
8953-UM
7301-m6A
7301-UM
6908-m6A
6908-UM
RNA pulldown
Input cell lysate
YTHDF3
G3BP1
YTHDF3
G3BP1
Relative band intensity (a.u.)
8953-m6A
8953-UM
7301-m6A
7301-UM
6908-m6A
6908-UM
0
15
30
45
0
10
20
30
40
50
60
0
5
10
15
20
25
30
35
40
45
50
YTHDF3
G3BP1

Fig. S10

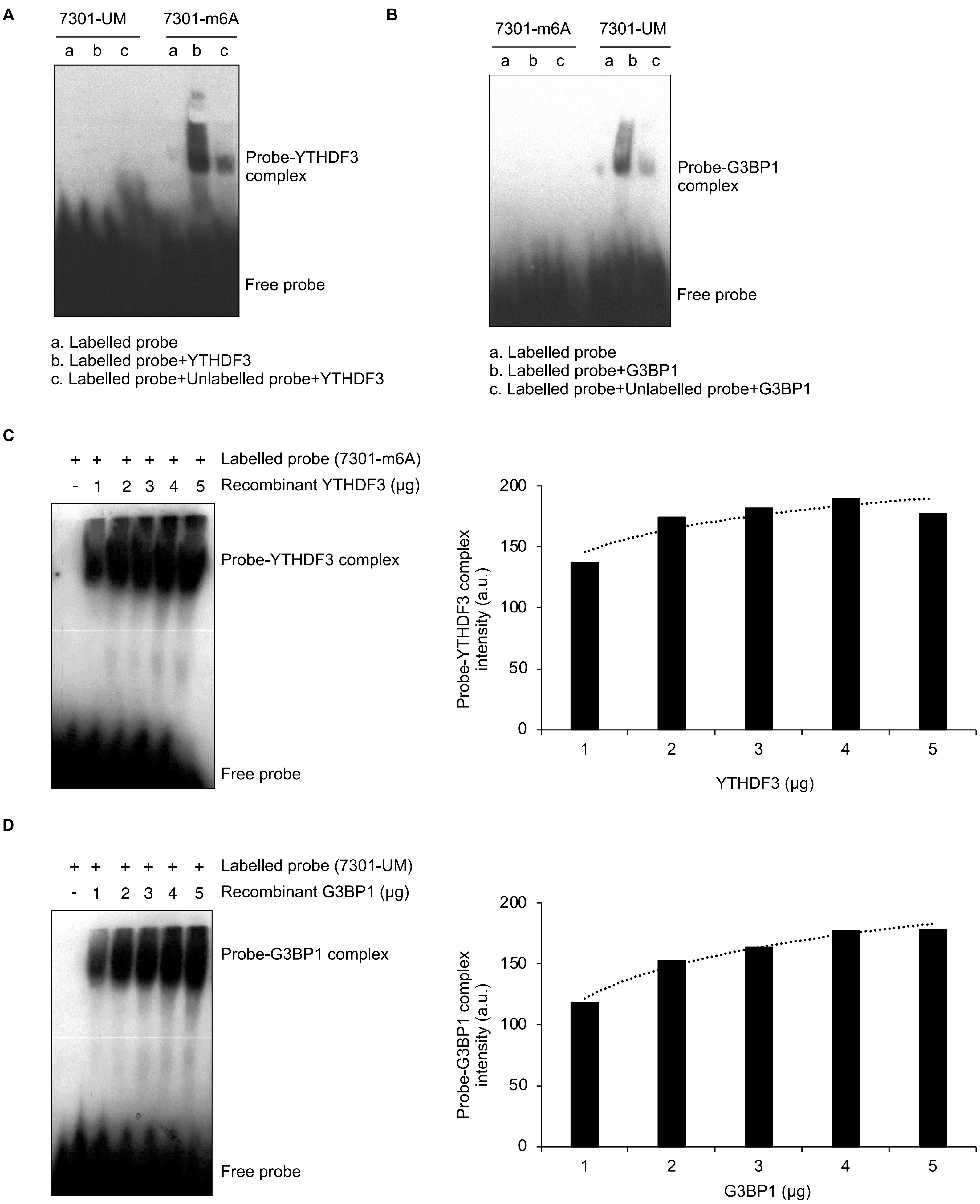
A
7301-UM
7301-m6A
a b c a b c
Probe-YTHDF3 complex
Free probe
a. Labelled probe
b. Labelled probe+YTHDF3
c. Labelled probe+Unlabelled probe+YTHDF3
B
7301-m6A
7301-UM
a b c a b c
Probe-G3BP1 complex
Free probe
a. Labelled probe
b. Labelled probe+G3BP1
c. Labelled probe+Unlabelled probe+G3BP1
C
+ + + + + + Labelled probe (7301-m6A)
- 1 2 3 4 5 Recombinant YTHDF3 (μg)
Probe-YTHDF3 complex
Free probe
Probe-YTHDF3 complex intensity (a.u.)
0
50
100
150
200
1 2 3 4 5
YTHDF3 (μg)
D
+ + + + + + Labelled probe (7301-UM)
- 1 2 3 4 5 Recombinant G3BP1 (μg)
Probe-G3BP1 complex
Free probe
Probe-G3BP1 complex intensity (a.u.)
0
50
100
150
200
1 2 3 4 5
G3BP1 (μg)

Fig. S11

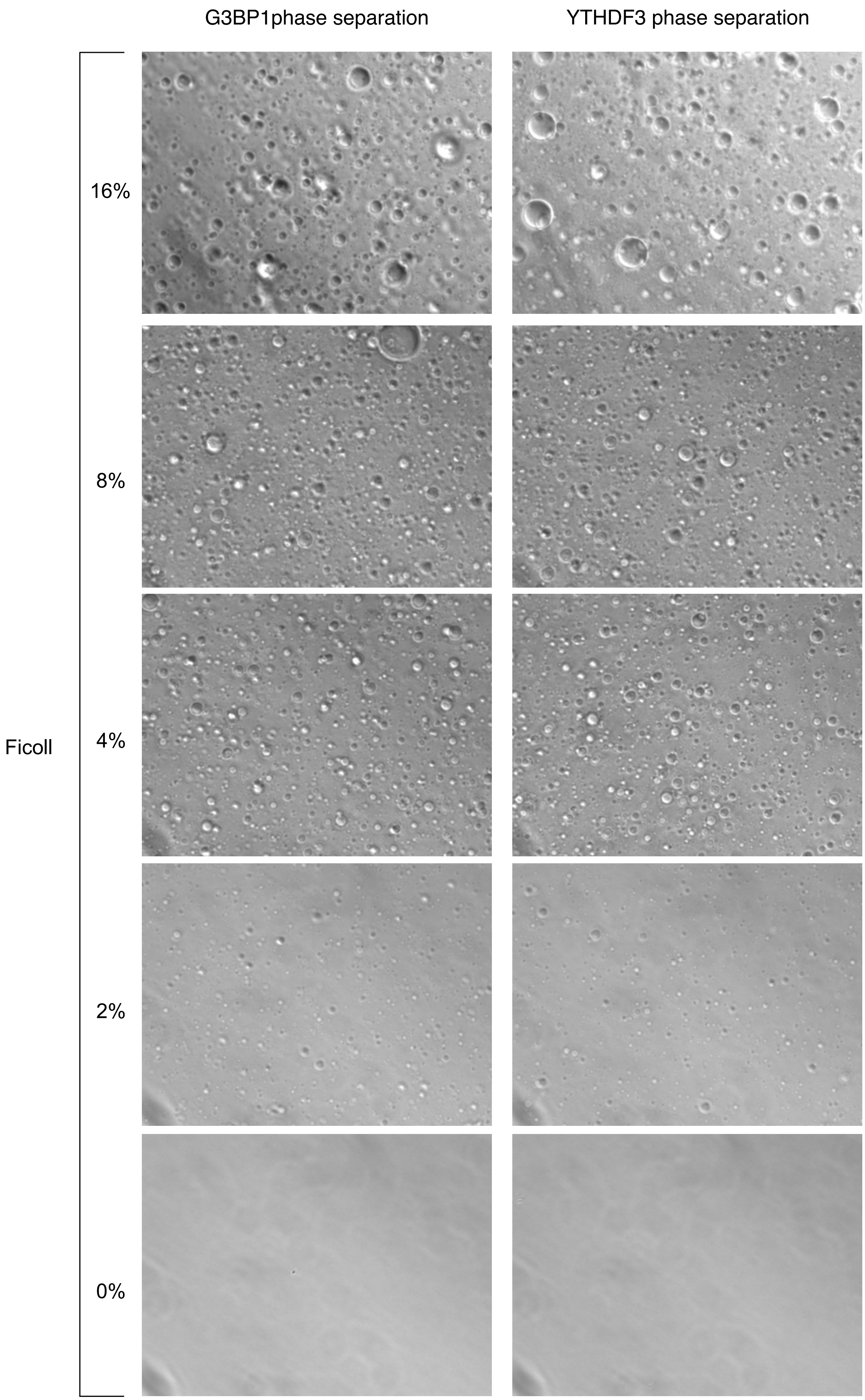

G3BP1phase separation
YTHDF3 phase separation
Ficoll
16%
8%
4%
2%
0%

Fig. S12

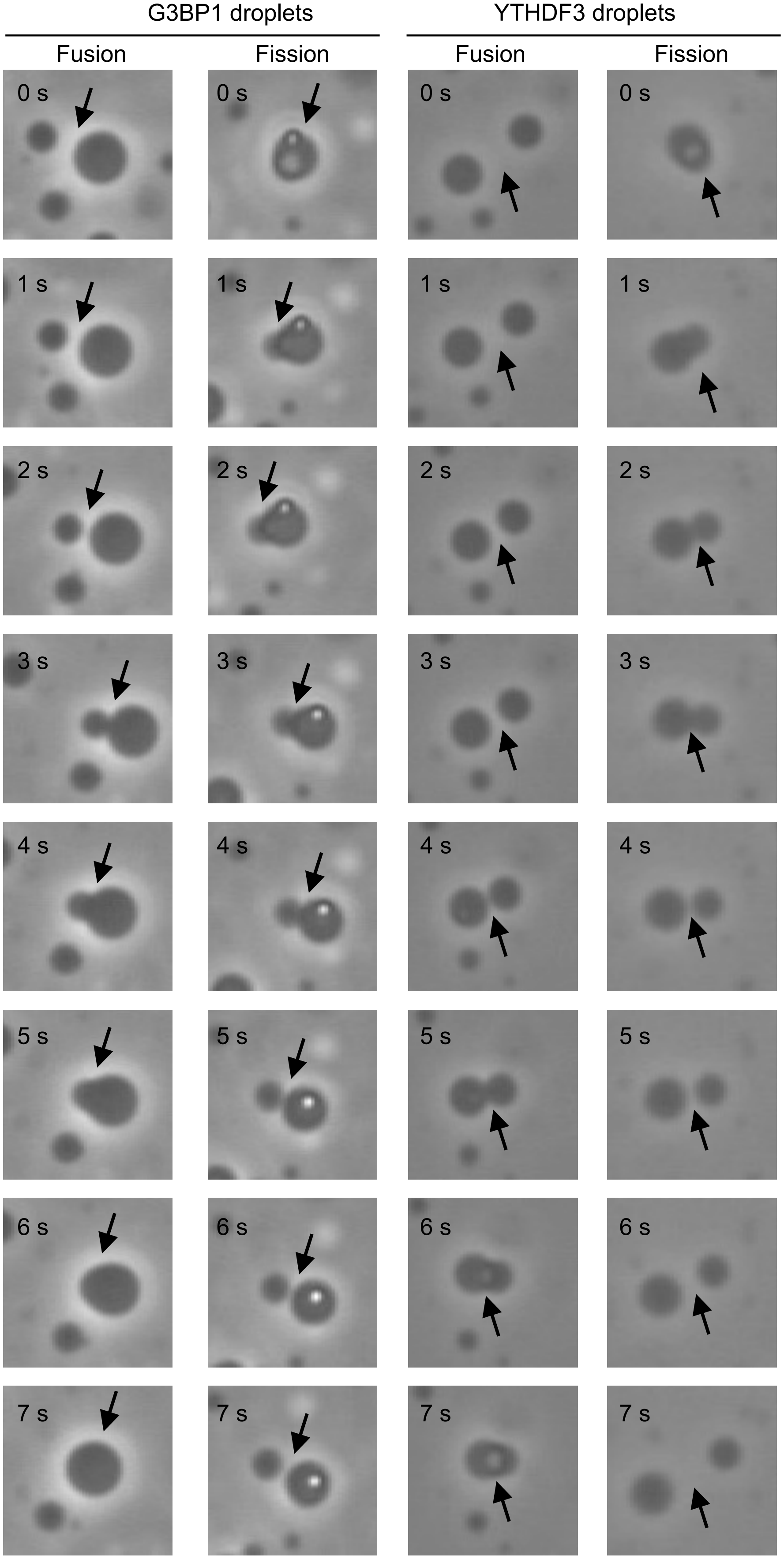
G3BP1 droplets
YTHDF3 droplets
Fusion
Fission
Fusion
Fission
0 s
1 s
2 s
3 s
4 s
5 s
6 s
7 s

Fig. S13

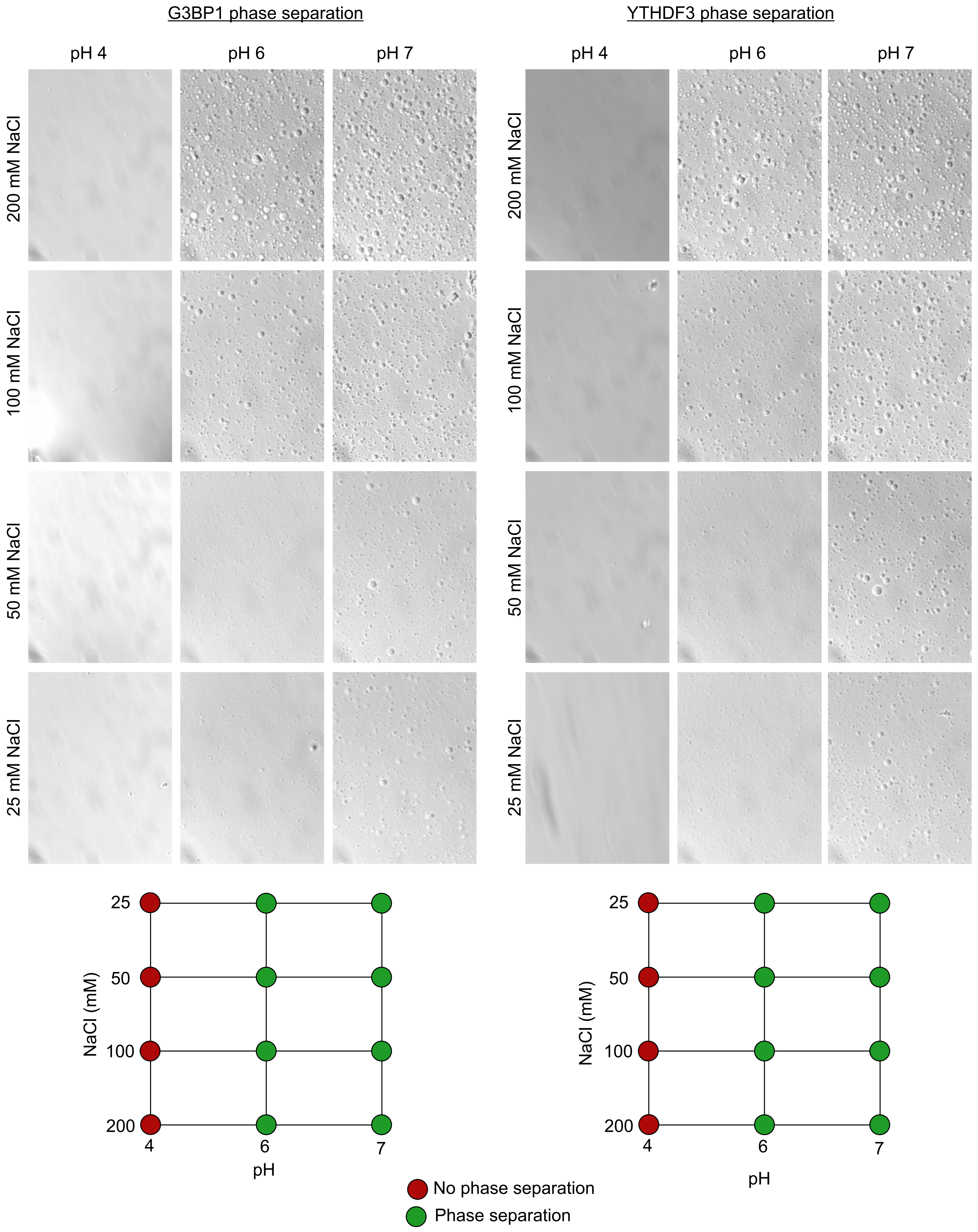
G3BP1 phase separation
YTHDF3 phase separation
pH 4
pH 6
pH 7
200 mM NaCl
100 mM NaCl
50 mM NaCl
25 mM NaCl
NaCl (mM)
25
50
100
200
pH
4
6
7
No phase separation
Phase separation

Fig. S14

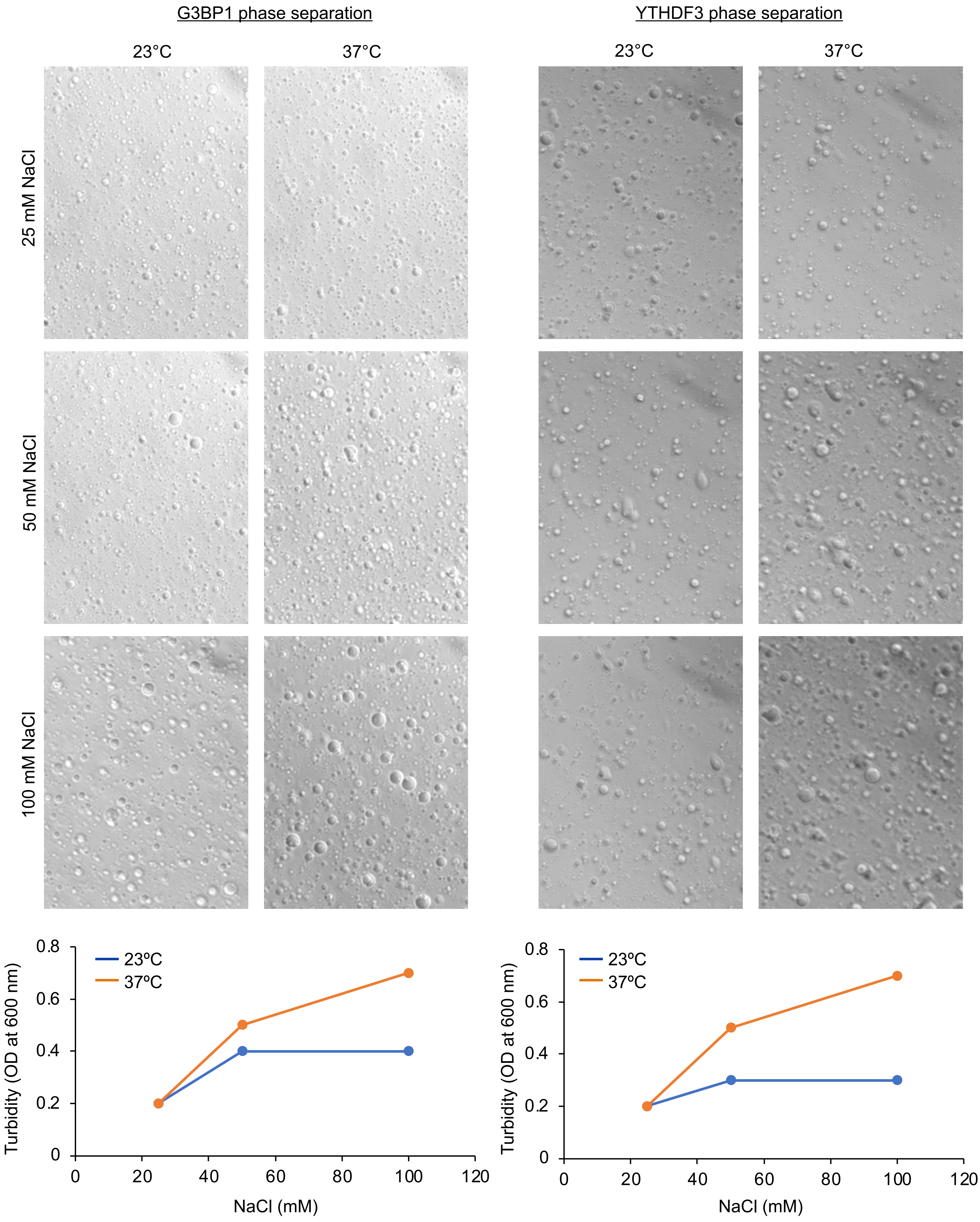
G3BP1 phase separation
YTHDF3 phase separation
23°C
37°C
23°C
37°C
25 mM NaCl
50 mM NaCl
100 mM NaCl
Turbidity (OD at 600 nm)
23ºC
37ºC
NaCl (mM)
0
0.2
0.4
0.6
0.8
0
20
40
60
80
100
120
Turbidity (OD at 600 nm)
23ºC
37ºC
NaCl (mM)

Fig. S15

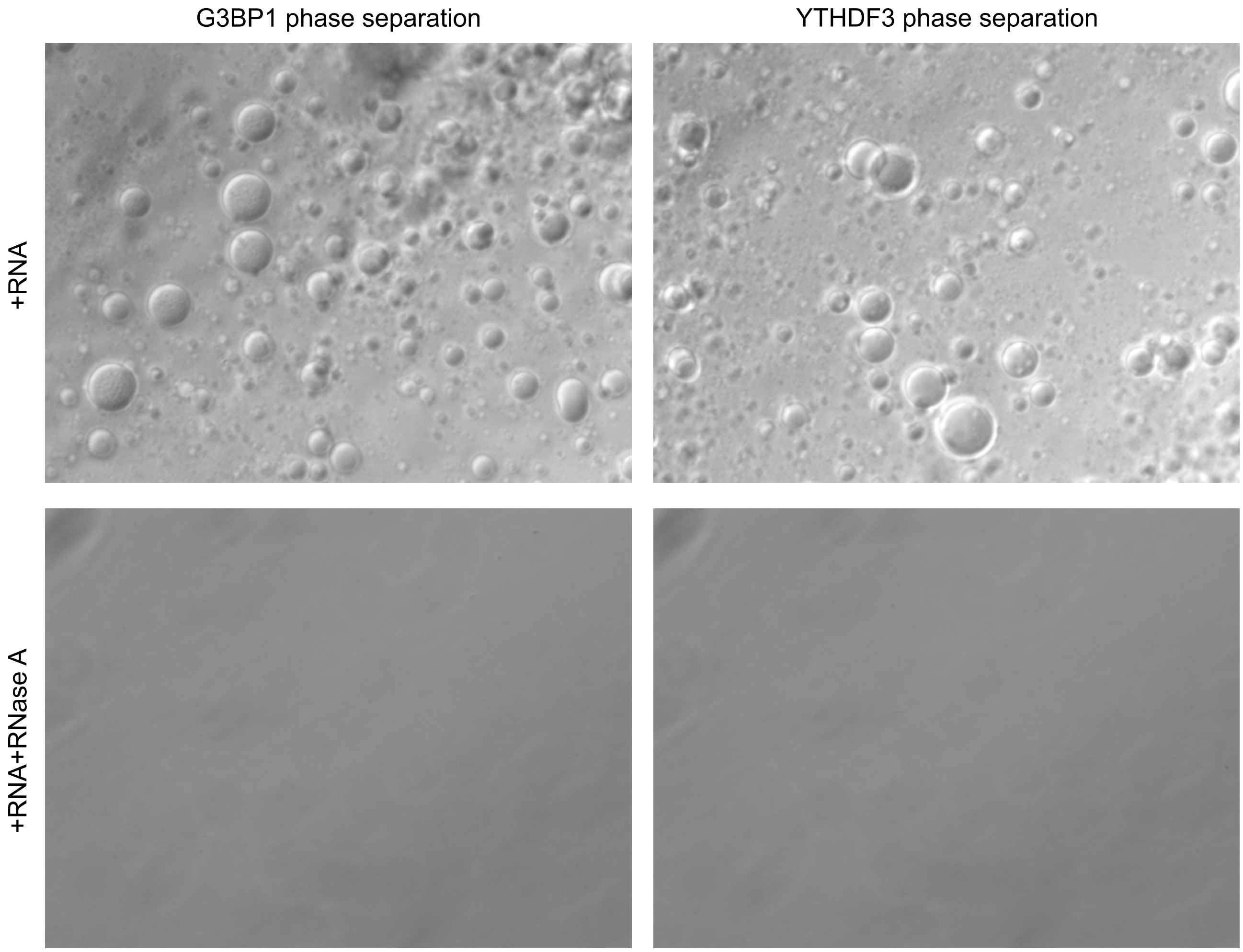
G3BP1 phase separation
YTHDF3 phase separation
+RNA
+RNA+RNase A

Fig. S16

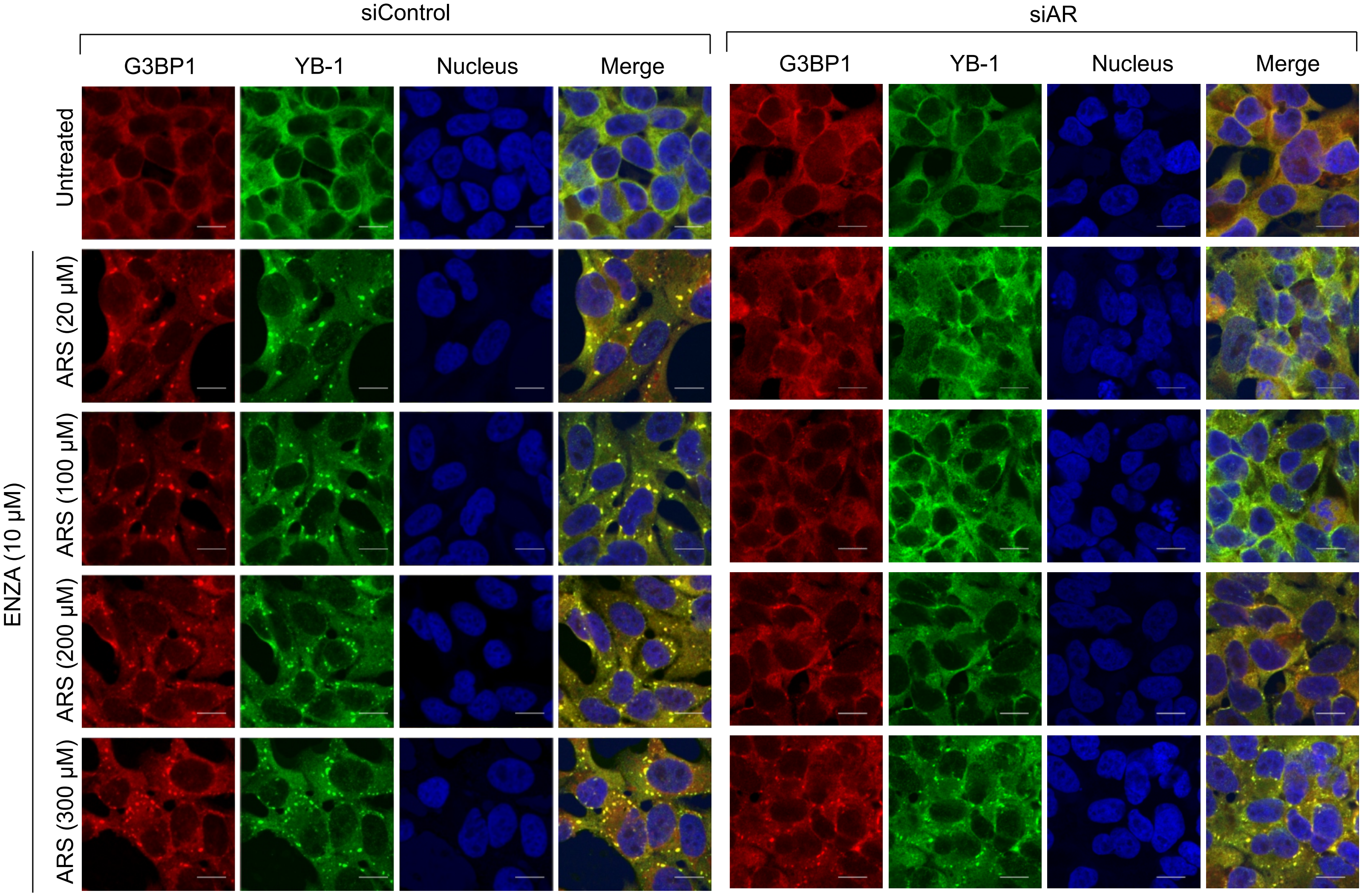

siControl
siAR
G3BP1
YB-1
Nucleus
Merge
G3BP1
YB-1
Nucleus
Merge
Untreated
ARS (20 μM)
ARS (100 μM)
ARS (200 μM)
ARS (300 μM)
ENZA (10 μM)

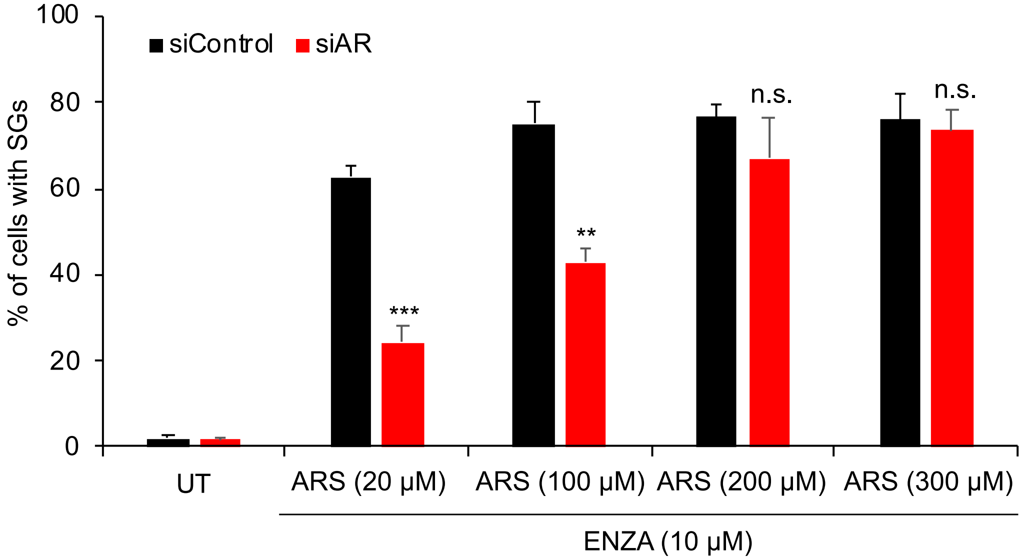

100
80
60
40
20
0
siControl
siAR
% of cells with SGs
***
**
n.s.
n.s.
UT
ARS (20 μM)
ARS (100 μM)
ARS (200 μM)
ARS (300 μM)
ENZA (10 μM)

Fig. S17

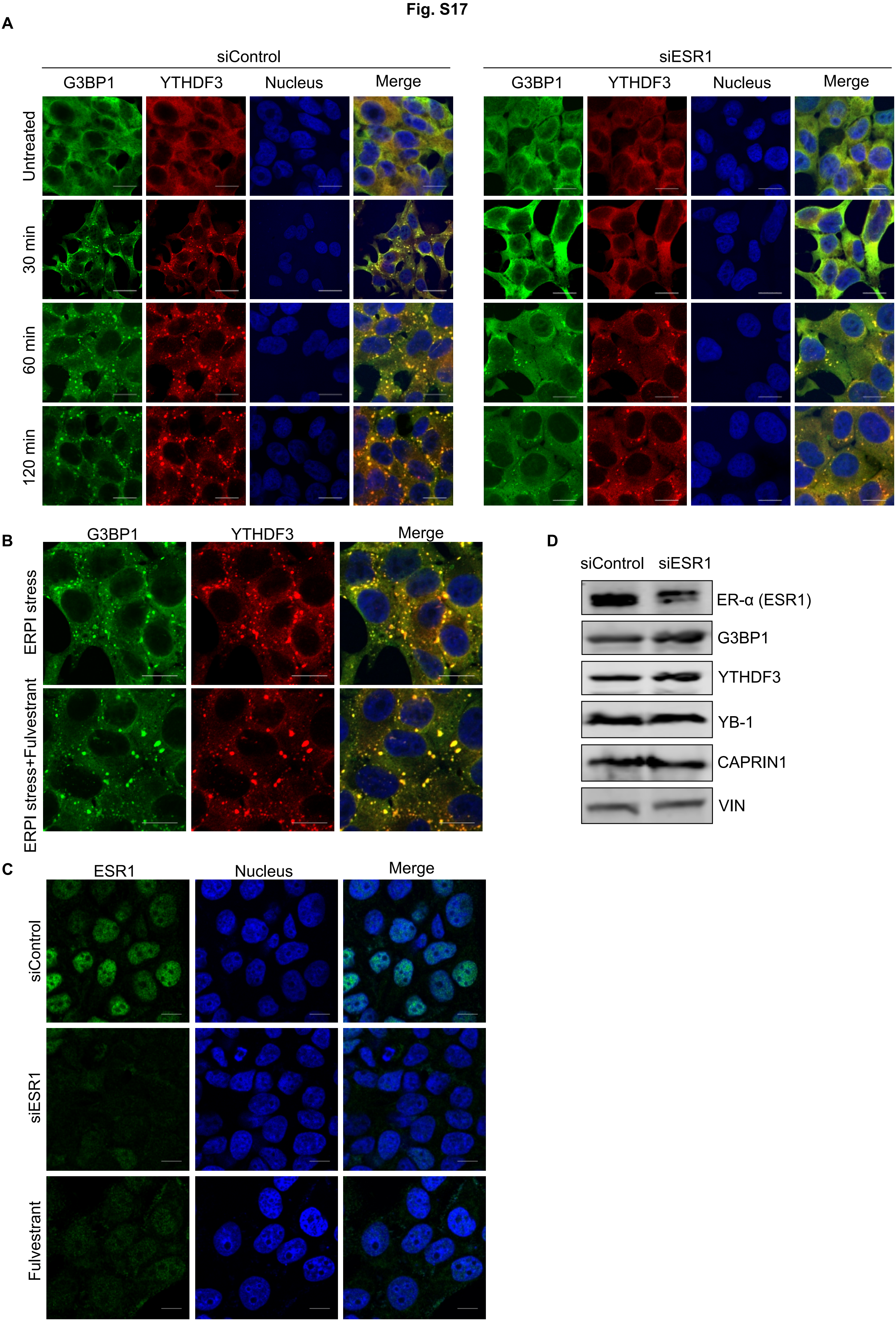
A
siControl
siESR1
G3BP1
YTHDF3
Nucleus
Merge
G3BP1
YTHDF3
Nucleus
Merge
Untreated
30 min
60 min
120 min
B
G3BP1
YTHDF3
Merge
ERPI stress
ERPI stress+Fulvestrant
C
ESR1
Nucleus
Merge
siControl
siESR1
Fulvestrant
D
siControl
siESR1
ER-α (ESR1)
G3BP1
YTHDF3
YB-1
CAPRIN1
VIN

Fig. S18

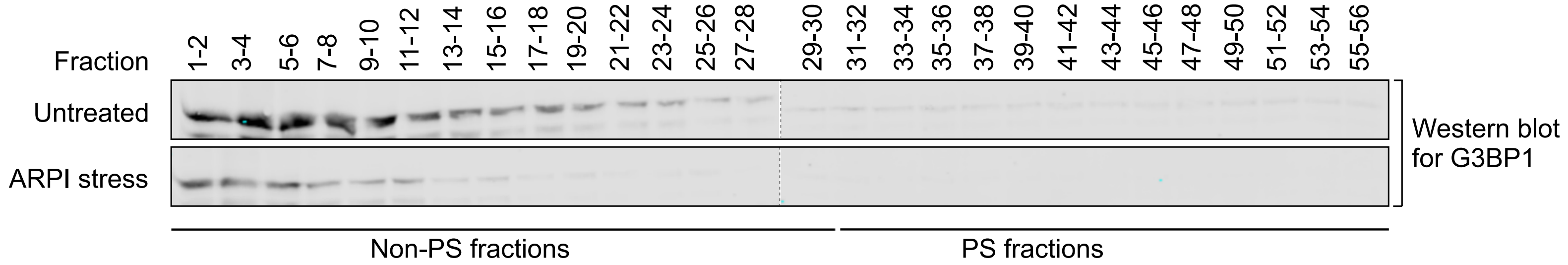

Fraction
1-2
3-4
5-6
7-8
9-10
11-12
13-14
15-16
17-18
19-20
21-22
23-24
25-26
27-28
29-30
31-32
33-34
35-36
37-38
39-40
41-42
43-44
45-46
47-48
49-50
51-52
53-54
55-56
Untreated
ARPI stress
Western blot for G3BP1
Non-PS fractions
PS fractions

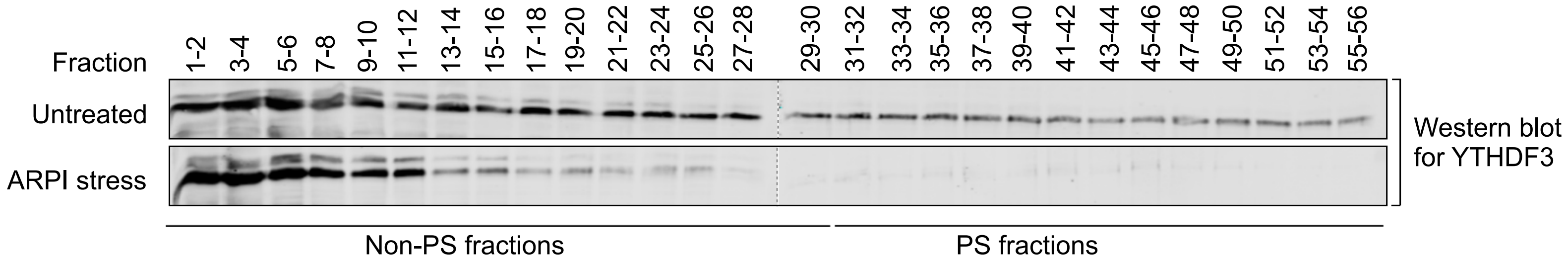

Fraction
1-2
3-4
5-6
7-8
9-10
11-12
13-14
15-16
17-18
19-20
21-22
23-24
25-26
27-28
29-30
31-32
33-34
35-36
37-38
39-40
41-42
43-44
45-46
47-48
49-50
51-52
53-54
55-56
Untreated
ARPI stress
Western blot for YTHDF3
Non-PS fractions
PS fractions

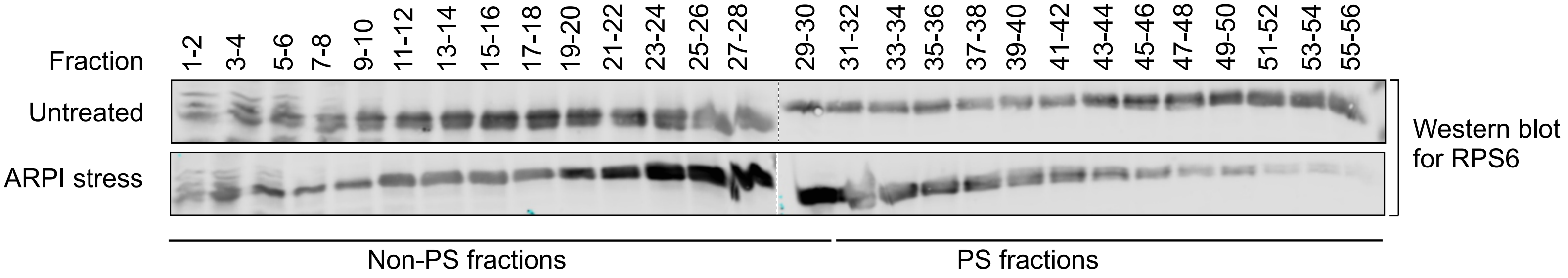

Fraction
1-2
3-4
5-6
7-8
9-10
11-12
13-14
15-16
17-18
19-20
21-22
23-24
25-26
27-28
29-30
31-32
33-34
35-36
37-38
39-40
41-42
43-44
45-46
47-48
49-50
51-52
53-54
55-56
Untreated
ARPI stress
Western blot for RPS6
Non-PS fractions
PS fractions

Fig. S19

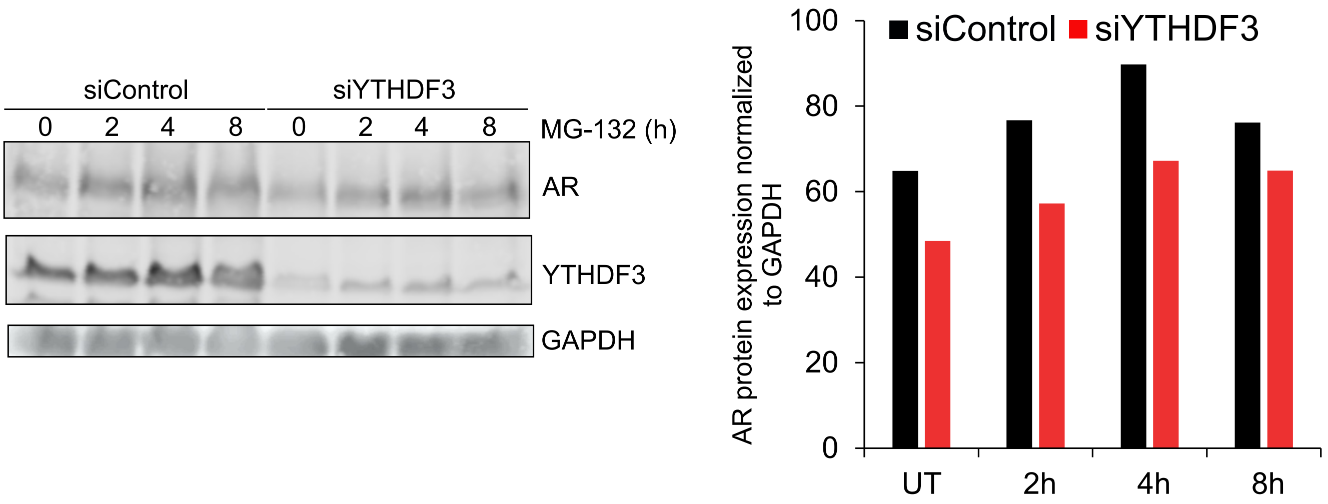

siControl
siYTHDF3
0
2
4
8
0
2
4
8
MG-132 (h)
AR
YTHDF3
GAPDH
siControl
siYTHDF3
AR protein expression normalized to GAPDH
100
80
60
40
20
0
UT
2h
4h
8h

Fig. S20

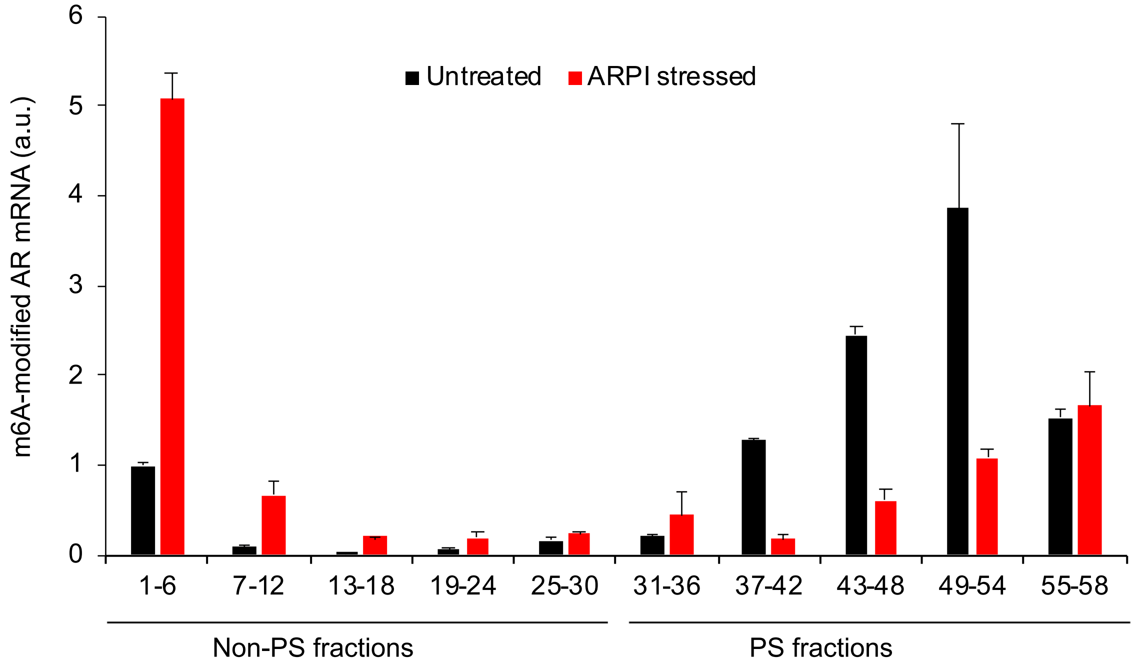

m6A-modified AR mRNA (a.u.)
0
1
2
3
4
5
6
Untreated
ARPI stressed
1-6
7-12
13-18
19-24
25-30
31-36
37-42
43-48
49-54
55-58
Non-PS fractions
PS fractions

Fig. S21

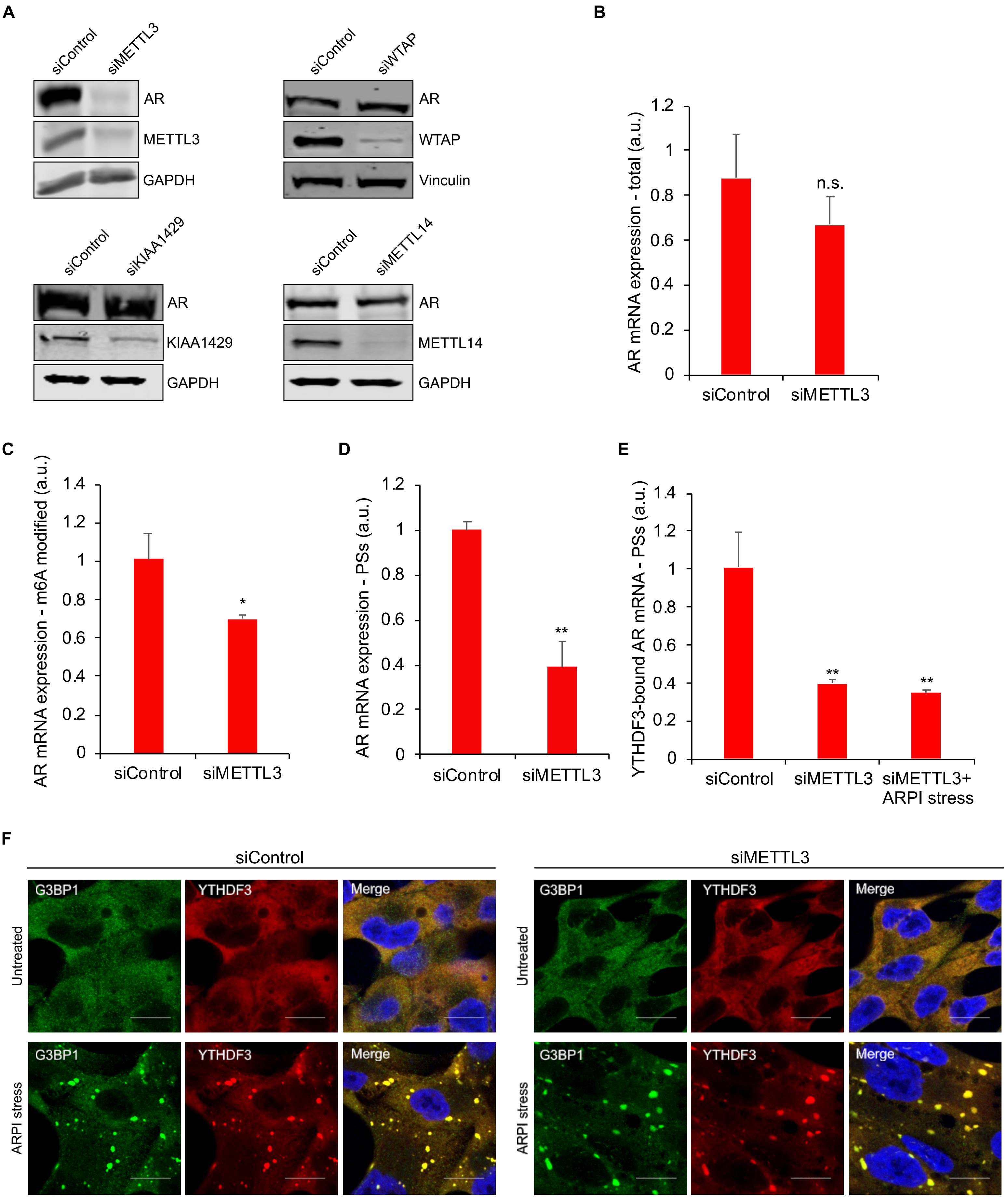
A
siControl
siMETTL3
AR
METTL3
GAPDH
siControl
siWTAP
AR
WTAP
Vinculin
siControl
siKIAA1429
AR
KIAA1429
GAPDH
siControl
siMETTL14
AR
METTL14
GAPDH
B
AR mRNA expression - total (a.u.)
1.2
1
0.8
0.6
0.4
0.2
0
n.s.
siControl
siMETTL3
C
AR mRNA expression - m6A modified (a.u.)
1.4
1.2
1
0.8
0.6
0.4
0.2
0
*
siControl
siMETTL3
D
AR mRNA expression - PSs (a.u.)
1.2
1
0.8
0.6
0.4
0.2
0
**
siControl
siMETTL3
E
YTHDF3-bound AR mRNA - PSs (a.u.)
1.4
1.2
1
0.8
0.6
0.4
0.2
0
**
**
siControl
siMETTL3
siMETTL3+ ARPI stress
F
siControl
siMETTL3
Untreated
ARPI stress
G3BP1
YTHDF3
Merge

Fig. S22

A

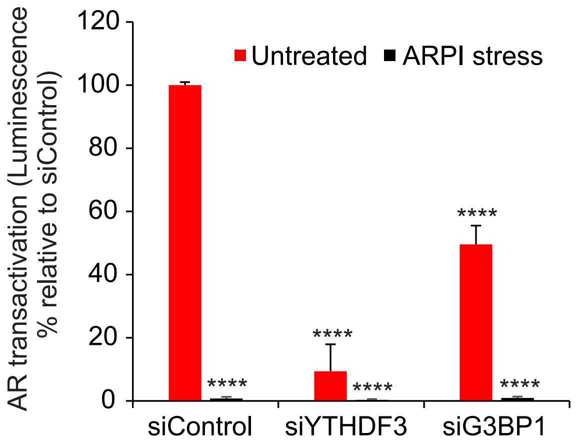
AR transactivation (Luminescence
% relative to siControl)
120
100
80
60
40
20
0
Untreated
ARPI stress
****
****
****
****
****
siControl
siYTHDF3
siG3BP1

B

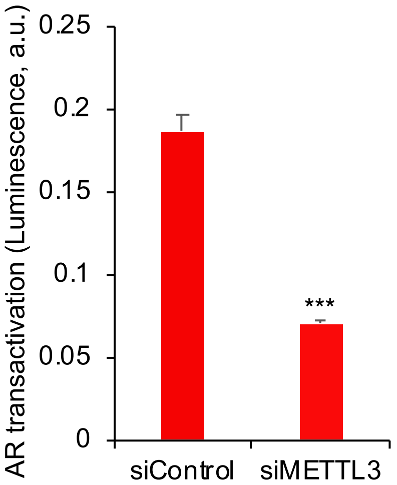
AR transactivation (Luminescence, a.u.)
0.25
0.2
0.15
0.1
0.05
0
***
siControl
siMETTL3

Fig. S23

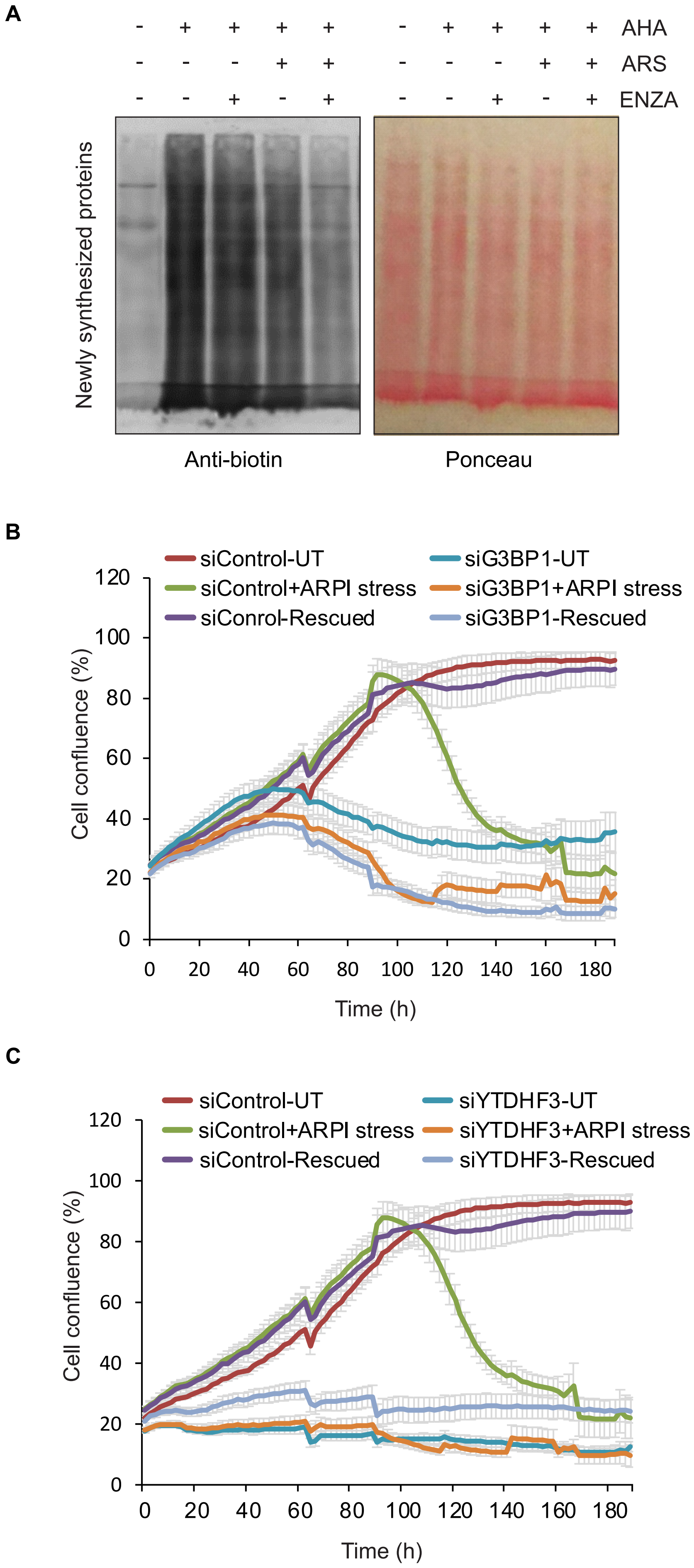
A
- + + + + - + + + + AHA
- - - + + - - - + + ARS
- - + - + - - + - + ENZA
Newly synthesized proteins
Anti-biotin
Ponceau
B
siControl-UT
siControl+ARPI stress
siConrol-Rescued
siG3BP1-UT
siG3BP1+ARPI stress
siG3BP1-Rescued
Cell confluence (%)
0 20 40 60 80 100 120
0 20 40 60 80 100 120 140 160 180
Time (h)
C
siControl-UT
siControl+ARPI stress
siControl-Rescued
siYTDHF3-UT
siYTDHF3+ARPI stress
siYTDHF3-Rescued
Cell confluence (%)
0 20 40 60 80 100 120
0 20 40 60 80 100 120 140 160 180
Time (h)

Supplementary Table 1

Oligos for EMSA

Oligos	Sequence
8953: m6A modified with biotin	5' Bi-AACUCAAUGGAm6ACUGACUGAGAUUUACCAC 3'
8953: m6A modified without biotin	5' AACUCAAUGGAm6ACUGACUGAGAUUUACCAC 3'
8953: Unmodified with biotin	5’ Bi-AACUCAAUGGAACUGACUGAGAUUUACCAC 3’
8953: Unmodified without biotin	5' AACUCAAUGGAACUGACUGAGAUUUACCAC 3’
6908: m6A modified with biotin	5' Bi-CCUGUGAGGGm6ACUGGCCACUCAGACCCACU 3'
6908: m6A modified without biotin	5' CCUGUGAGGGm6ACUGGCCACUCAGACCCACU 3'
6908: Unmodified with biotin	5’ Bi-CCUGUGAGGGACUGGCCACUCAGACCCACU 3’
6908: Unmodified without biotin	5’ CCUGUGAGGGACUGGCCACUCAGACCCACU 3’
7301: m6A modified with biotin	5' Bi-GGAUAUUUUGAAGGm6ACUGUCAUAUAUCUUU 3'
7301: m6A modified without biotin	5' GGAUAUUUUGAAGGm6ACUGUCAUAUAUCUUU 3'
7301: Unmodified with biotin	5' Bi-GGAUAUUUUGAAGGACUGUCAUAUAUCUUU 3'
7301: Unmodified without biotin	5' GGAUAUUUUGAAGGACUGUCAUAUAUCUUU 3'

Oligos for phase separation

Oligos	Sequence
8953: m6A modified with FITC	5' Fl-AACUCAAUGGAm6ACUGACUGAGAUUUACCAC 3'
8953: Unmodified with FITC	5’ Fl-AACUCAAUGGAACUGACUGAGAUUUACCAC 3’

Oligos for qRT-PCR

Oligos	Sequence
ACC# NM_000044.6 FWD Set 1 (AR)	5’ GGTTACACCAAAGGGCTAGAA 3’
ACC# NM_000044.6 REV Set 1 (AR)	5’ GACTTGTAGAGAGACAGGGTAGA 3’
ACC# NM_000044.6 FWD Set 2 (AR)	5’ CTTCCCATTGTGGCTCCTATC 3’
ACC# NM_000044.6 REV Set 2 (AR)	5’ GGCACAGAGTAGTGCTGTAAA 3’
ACC# NR_145820.1 FWD Set 1 (18S RNA)	5’ ACGTCTGCCCTATCAACTTTC 3’
ACC# NR_145820.1 REV Set 1 (18S RNA)	5’ CCGCGGTCCTATTCCATTATT 3’
ACC# NR_145820.1 FWD Set 2 (18S RNA)	5’ GGTTCGAAGACGATCAGATACC 3’
ACC# NR_145820.1 REV Set 2 (18S RNA)	5’ GCTTATGACCCGCACTTACT 3’
4331182 Hs02786624_g1 GAPDH	Commercial probe from Thermo Fisher Scientific
4331182 Hs00171172_m1 AR	Commercial probe from Thermo Fisher Scientific
