# Supplementary material for: Regulation of AR mRNA translation in response to acute AR pathway inhibition
Source: Nucleic Acids Res. 2021 Dec 23;50(2):1069–91. doi: 10.1093/nar/gkab1247 (PMC8789049; doi:10.1093/nar/gkab1247)
Supplement: gkab1247_Supplemental_Files [file gkab1247_supplemental_files.zip › Supplementary Figure legends NAR-03196-V-2021.pdf]

SUPPLEMENTARY FIGURE LEGENDS

Figure S1. ARPI stress induces SG formation in LNCaP cells. (**A**) LNCaP cells were unstressed or pre-treated with ENZA for 24 h and followed by treatment with arsenite (ARS), thapsigargin (TGN) or sorbitol (SBL). The cells were stained with anti-G3BP1 and anti-YB-1 antibodies (left-side panel). LNCaP cells were pre-treated with ODM-201 (Darolutamide), VPC-14449 or EPI-001 for 24 h and followed by treatment with ARS for 1 h. The cells were stained with anti-G3BP1 and anti-YB-1 antibodies (right-side upper panel). Quantification of SGs is shown in the right-side bottom panel. Note that ARPI stress induces SGs while ARPI or individual stresses alone do not induce SGs. The results are an average of three independent experiments with p***<0.001. Scale 10 μm.

Figure S2. ARPI stress induces SG formation in V-16-D cells and not in PC-3 cells. (**A**) V-16-D cells were unstressed or pre-treated with ENZA for 24 h, and followed by treatment with arsenite (ARS) and thapsigargin (TGN) for 1 h. The cells were stained with anti-G3BP1 and anti-YB-1 antibodies. Quantification of SGs shown in the left-side bottom panel. Note that ARPI stress induces SGs, while ARPI or individual stresses alone do not induce SGs. **(B)** PC-3 cells were unstressed or stressed with ENZA for 24 h. The cells were then stressed with different concentrations of ARS (20 μM to 160 μM) and subjected to IF (immunofluorescence) using anti-G3BP1 and anti-YTHDF3 antibodies. Quantification of SGs is shown on the right-side bottom panel. Note that SGs were not formed with ARS alone or ENZA+ARS at 20 or 80 μM ARS concentrations. SGs were formed at an equal rate in cells stressed with 160 μM ARS or ENZA+160 μM ARS, indicating that ENZA treatment did not have an additional effect on SG formation compared to treatment with ARS alone. The results are an average of three independent experiments with p***<0.001. n.s, non-significant. Scale 10 μm.

Figure S3. Anti-cancer therapeutics other than ARPI do not induce SGs in combination with low dose of arsenite. (**A**) LNCaP cells were untreated or treated with the indicated concentrations of ARPI drug bicalutamide (Bic), microtubule inhibitor docetaxel (Doce) or PARP inhibitor olaparib (Olap) for 24 h followed by treatment with arsenite (ARS) for 1 h. The cells were stained with anti-G3BP1 and anti-YTHDF3 antibodies. Quantification of SGs is shown in the right-side bottom panel. Note that while bicalutamide in combination with arsenite induces SGs, docetaxel and olaparib did

not induce SGs in combination with arsenite. Scale 10 μm. (**B)** Treatment with docetaxel or olaparib significantly reduced the survival of cells as measured by IncuCyte® Cell Count Proliferation Assay.

Figure S4. ARPI stress did not affect total AR mRNA and the stability of AR protein. (**A**) qRT-PCR showing total AR mRNA levels are not affected by ARPI stress. The results are an average of three independent experiments. n.s, non-significant. (**B**) LNCaP cells were stressed with ENZA (10 μM) for 24 hours. MG132 (10 μM) or Chloroquine (CQ, 10 μg/ml) were applied to the cells for 6 h after the start of ENZA treatment. Arsenite (ARS, 20 μM) was included during the last hour of the treatment. Protein lysates were subjected to Western blotting for AR. Vinculin is used as a loading control.

Figure S5. The percentage of AR mRNA across the gradient in untreated and in ARPI-stressed cells. The relative distribution of AR mRNA across the gradient using continuous measurements on a single graph is provided. For normalisation, the relative levels in the various fractions across the gradient is added up to 100. Note that AR mRNA is redistributed from PSs in untreated cells to non-PS fractions in ARPI stressed cells.

Figure S6. In situ hybridisation (ISH) of AR mRNA. (**A**) LNCaP cells transfected with siControl or siAR siRNAs were untreated or treated with ARPI stress. The cells were subjected to in situ hybridisation (ISH) using AR specific probe. Note that siControl cells showed a strong ISH signal represented by red spots, while the siAR cells did not show any detectable signal, showing the specificity of the AR mRNA probe. (**B**) LNCaP cells were subjected to ISH following the same hybridisation conditions as above with a negative control probe targeting the DapB gene (accession # EF191515) from the Bacillus subtilis strain SMY, a soil bacterium. Note that hybridisation with the DapB probe did not give a detectable signal, excluding any non-specific binding in the ISH conditions. Scale 10 μm.

Figure S7. Purification of recombinant proteins. PAGE analysis showing recombinant proteins purified through Ni-NTA affinity chromatography. (A) YTHDF3, (B) G3BP1, (C) GFP-YTHDF3, and (D) RFP-G3BP1.

Figure S8. Titration of m6A modified or unmodified RNA probes with YTHDF3 or G3BP1 recombinant proteins using EMSA. Biotin-labelled RNA probes (8953-m6A or 6908-m6A) were incubated with increasing concentration of recombinant YTHDF3 (**A**), or biotin-labelled unmodified RNA probes (8953-UM or 6908-UM) were incubated with increasing concentrations of recombinant G3BP1 (**B**), and subjected to EMSA. The mobility shift of probe-protein complex and free probe are indicated. Histograms represent the quantification of the intensity of the bands corresponding to the probe-protein complexes.

Figure S9. RNA affinity chromatography. Biotin-labelled m6A modified or unmodified AR mRNA probes incubated with lysates from LNCaP cells were pulldown using Streptavidin beads and subjected to Western blotting for YTHDF3 and G3BP1. Quantification of blots are provided in the bottom panel.

Figure S10. RNA EMSA to analyse the direct binding of YTHDF3 or G3BP1 to m6A-modified or unmodified probes corresponding to region 7301 of AR mRNA. Biotin-labelled probes were mixed with recombinant YTHDF3 (**A**) or G3BP1 (**B**) and subjected to EMSA. A probe mobility shift in the presence of YTHDF3 or G3BP1 and free probe are indicated. A 200-fold molar excess concentration of unlabeled probes was added along with the labelled probe to demonstrate the specificity of RNA-protein complex formation. Biotin-labelled m6A-modified RNA probe (7301-m6A) was incubated with increasing concentration of recombinant YTHDF3 (**C**), and biotin-labelled unmodified RNA probe (7301-UM) was incubated with increasing concentration of G3BP1 (**D**) and subjected to EMSA. The mobility shift of probe-protein complex and free probe are indicated. Histograms on the right-side panels represent quantification of the intensity of bands corresponding to the probe-protein complex.

Figure S11. Phase separation G3BP1 and YTHDF3. Recombinant G3BP1 or YTHDF3 was incubated with different concentrations of Ficoll (0-16%) to induce phase separation. The phase-separated samples were analysed by a phase contrast microscope at 32x.

Figure S12. Fusion and fission of G3BP1 and YTHDF3 droplets. Time-lapse images showing the droplet fusion and fission of phase-separated G3BP1 and YTHDF3.

Figure S13. Salt and pH sensitivity of G3BP1 and YTHDF3 phase separation. Recombinant G3BP1 or YTHDF3 were subjected to phase separation in buffer containing different concentrations of NaCl or at different pH. A summary of phase separation behaviours of G3BP1 or YTHDF3 under these conditions is provided in the bottom panels.

Figure S14. Temperature sensitivity of G3BP1 and YTHDF3 phase separation. Recombinant G3BP1 or YTHDF3 were subjected to phase separation in buffer containing different concentrations of NaCl at 23ºC or 37ºC. The efficiency of phase separation quantified by measuring the turbidity of the suspension at OD^{600} is provided in the bottom panels.

Figure S15. RNA is required for the phase-separation of G3BP1 and YTHDF3. Recombinant G3BP1 or YTHDF3 were mixed with RNA -/+RNase A, and subjected to phase-separation to form the liquid droplets. Note that treatment with RNase A completely prevented the formation of droplets for both G3BP1 and YTHDF3.

Figure S16. AR KD using siRNAs delayed the formation of SGs. LNCaP cells transfected with siControl or siAR siRNAs were subjected to -/+ ARPI stress [10 μM ENZA (24 h) and different concentrations of ARS such as 20 μM, 100 μM, 200 μM, and 300 μM for 1h]. The cells were stained with anti-G3BP1 and anti-YB-1 antibodies to analyse SGs. Quantification of SGs is provided in the bottom panel. Note that SG formation is significantly delayed at 20 μM and 100 μM ARS, while this effect is gradually lost at 200 μM and 300 μM ARS treatment. The results are an average of three independent experiments with p***<0.001, p**<0.01. n.s, non-significant. Scale 10 μm.

Figure S17. ESR1 at the protein level did not affect SG formation. (**A**) Knockdown of ESR1 delayed the formation of SGs after ERPI (estrogen receptor pathway inhibition) stress in MCF7 cells. MCF7 cells transfected with siControl or siESR1 siRNAs were unstressed or stressed with ERPI stress for 30 min, 60 min and 120 min, and stained with anti-G3BP1 and anti-YTHDF3 antibodies to reveal the SGs. Note that SGs are formed after ERPI stress in siControl cells, while SG formation is significantly delayed at 30 min and 60 min with a reduced effect was observed at 120 min of treatment in siESR1 cells compared to siControl cells. (**B**) Reducing the protein level of ESR1 using fulvestrant treatment did not affect the formation of SGs after ERPI stress in MCF7 cells. (**C**) IF images

demonstrating the downregulation of ESR1 in siESR1 and fulvestrant treated cells compared control cells. (**D**) Knockdown of ER-α in MCF7 cells did not affect the protein level of G3BP1, YTHDF3, YB-1, and CAPRIN1. Vinculin (VIN) is used as the loading control. Scale 10 μm.

Figure S18. Distribution of G3BP1 (top panel) and YTHDF3 (middle panel) in the different sucrose gradient fractions. Note that G3BP1 is present mainly in the non-PS fractions in untreated and ARPI stressed cells. YTHDF3 is distributed in the PS and non-PS fractions in untreated cells. After ARPI stress, YTHDF3 redistributed from the PS fractions to non-PS fractions. RPS6 is used as a loading control (bottom panel).

Figure S19. Stability of AR after YTHDF3 knockdown. LNCaP cells were transfected with siControl or siYTHDF3 siRNAs. The cells were then untreated or treated with proteasome inhibitor MG-132 for the indicated time points, and cell lysates were subjected to Western blotting using anti-AR and anti-YTHDF3 antibodies. GAPDH was used as the loading control. The bands corresponding to AR were quantitated and shown on the right-side panel.

Figure S20. m6A modified AR mRNA is enriched in the PSs. The relative distribution of m6A-modified AR mRNA across the sucrose gradient in untreated and ARPI stressed cells. Note that m6A-modified AR mRNA is mainly distributed in the PSs in untreated cells. After ARPI stress, m6A modified AR mRNA is redistributed from the PSs to untranslatable non-PS fractions.

Figure S21. KD of METTL3 reduced the level of AR protein. (**A**) LNCaP cells transfected with siControl, siMETTL3, siWTAP, siKIAA1429 (VIRMA), or siMETTL14 cells were subjected to Western blotting with anti-AR, anti-METTL3, anti-WTAP, anti-KIAA1429 (VIRMA), anti-METTL14 antibodies. Note that KD of METTL3 significantly reduced the level of AR protein while the KD of WTAP, KIAA1429 or METTL3 did not affect the protein level of AR. GAPDH and Vinculin were used as the loading controls. (**B**) qRT-PCR showing no change in the expression of AR mRNA in METTL3 KD cells compared to control cells. (**C**) qRT-PCR is showing a reduction in the expression of m6A modified AR mRNA in total RNA from METTL3 KD cells compared to siControl cells. (**D**) qRT-PCR showing a reduction in the expression of m6A-modified AR mRNA in PSs from METTL3 KD cells compared to control cells. (**E**) qRT-PCR showing a reduction in the interaction of AR mRNA with YTHDF3 in the PSs of siMETTL3 -/+ ARPI stressed cells compared control

cells. The results are an average of three independent experiments with p**<0.01, p*<0.05, n.s., non-significant. (**F**) METTL3 KD did not affect the formation of SGs in response to ARPI stress. LNCaP cells transfected with siControl or siMETTL3 siRNA were untreated or treated with ARPI stress and subjected to immunostaining using anti-G3BP1 and anti-YTHDF3 antibodies. Scale 10 μm.

Figure S22. Effect of YTHDF3, G3BP1 and METTL3 on AR activity. (**A**) YTHDF3 or G3BP1 KD decreases AR transactivation. (**B**) METTL3 KD decreases AR transactivation. The results are presented as an average of 3 independent experiments with 24 replicates for each condition. p***<0.001, ****p<0.0001.

Figure S23. Effect of ARPI stress on global protein synthesis and proliferation of PCA cells. **(A)** ARPI stress reduced overall protein synthesis. Cell lysates prepared from AHA-labelled unstressed and ARPI stressed cells were subjected to Click reactions with biotin alkyne and immunoblotted with anti-biotin antibodies. Equal loading was determined by Ponceau staining shown on the right-side panel. **(B** and **C**) Real time cell proliferation assay in LNCaP cells showing reduced cell growth after ARPI stress. This effect was more pronounced when G3BP1 (**B**) or YTDHF3 (**C**) was silenced in the cells. The detrimental effect of ARPI stress on the growth of LNCaP cells was rescued once cells were exposed to fresh media. However, G3BP1 or YTDHF3 silencing failed to restore the cell growth.
